# Large-scale genomic analysis shows association between homoplastic genetic variation in *Mycobacterium tuberculosis* genes and meningeal or pulmonary tuberculosis

**DOI:** 10.1186/s12864-018-4498-z

**Published:** 2018-02-05

**Authors:** Carolien Ruesen, Lidya Chaidir, Arjan van Laarhoven, Sofiati Dian, Ahmad Rizal Ganiem, Hanna Nebenzahl-Guimaraes, Martijn A. Huynen, Bachti Alisjahbana, Bas E. Dutilh, Reinout van Crevel

**Affiliations:** 1 Department of Internal Medicine, Radboud university medical center, Nijmegen, The Netherlands; 2 Health Research Unit, Faculty of Medicine, Padjadjaran University/Hasan Sadikin Hospital, Bandung, Indonesia; 3 Department of Neurology, Faculty of Medicine, Padjadjaran University/Hasan Sadikin Hospital, Bandung, Indonesia; 4 National Institute for Public Health and the Environment (RIVM), Bilthoven, The Netherlands; 5 Life and Health Sciences Research Institute (ICVS), School of Health Sciences, University of Minho, Braga, Portugal; 6 ICVS/3B’s, PT Government Associate Laboratory, Braga, Portugal; 7 Centre for Molecular and Biomolecular Informatics, Radboud university medical center, Nijmegen, The Netherlands; 8 Theoretical Biology and Bioinformatics, Science4Life, Utrecht University, Utrecht, The Netherlands

**Keywords:** Pulmonary tuberculosis, Tuberculous meningitis, Whole genome sequencing, Homoplasy

## Abstract

**Background:**

Meningitis is the most severe manifestation of tuberculosis. It is largely unknown why some people develop pulmonary TB (PTB) and others TB meningitis (TBM); we examined if the genetic background of infecting *M. tuberculosis* strains may be relevant.

**Methods:**

We whole-genome sequenced *M. tuberculosis* strains isolated from 322 HIV-negative tuberculosis patients from Indonesia and compared isolates from patients with TBM (*n* = 106) and PTB (*n* = 216). Using a phylogeny-adjusted genome-wide association method to count homoplasy events we examined phenotype-related changes at specific loci or genes in parallel branches of the phylogenetic tree. Enrichment scores for the TB phenotype were calculated on single nucleotide polymorphism (SNP), gene, and pathway level. Genetic associations were validated in an independent set of isolates.

**Results:**

Strains belonged to the East-Asian lineage (36.0%), Euro-American lineage (61.5%), and Indo-Oceanic lineage (2.5%). We found no association between lineage and phenotype (Chi-square = 4.556; *p* = 0.207). Large genomic differences were observed between isolates; the minimum pairwise genetic distance varied from 17 to 689 SNPs. Using the phylogenetic tree, based on 28,544 common variable positions, we selected 54 TBM and 54 PTB isolates in terminal branch sets with distinct phenotypes. Genetic variation in Rv0218, and absence of Rv3343c, and *nanK* were significantly associated with disease phenotype in these terminal branch sets, and confirmed in the validation set of 214 unpaired isolates.

**Conclusions:**

Using homoplasy counting we identified genetic variation in three separate genes to be associated with the TB phenotype, including one (Rv0218) which encodes a secreted protein that could play a role in host-pathogen interaction by altering pathogen recognition or acting as virulence effector.

**Electronic supplementary material:**

The online version of this article (10.1186/s12864-018-4498-z) contains supplementary material, which is available to authorized users.

## Background

Tuberculosis (TB), caused by *Mycobacterium tuberculosis*, remains a major global health problem [1]. Active TB mostly affects the lungs but may also spread to other organs. TB meningitis (TBM), which represents approximately 1–5% of all TB cases, is the most severe manifestation of TB, resulting in death or neurological disability in about half of those affected [2, 3]. It is largely unknown why certain people develop pulmonary TB (PTB) and others TBM. Host immune-related factors clearly play an important role, as shown by the increased risk of TBM for patients with advanced HIV infection, and the overrepresentation of young children among TBM patients. Host genetic factors may also play a role; single studies have linked susceptibility to TBM with variation in candidate genes [4–8].

Besides the host, genetic diversity of infecting *M. tuberculosis* strains may also affect disease phenotype. Even though *M. tuberculosis* is considered a clonal organism, there is considerable genetic variation in the genomes of infecting *M. tuberculosis* isolates [9, 10]. Epidemiological studies have reported significant differences among *M. tuberculosis* lineages in terms of virulence [11, 12], transmission [9, 13, 14], progression to active disease after infection [15], and response to treatment [16, 17]. In vitro studies have supported these findings by showing *M. tuberculosis* genotype-specific differences in the human immune response [18–21].

Animal studies have shown that *M. tuberculosis* strains differ in their ability to invade the central nervous system (CNS). Five *M. tuberculosis* genes (Rv0311 (unknown function), Rv0805 (intermediary metabolism and respiration), *pknD* (protein kinase D), Rv0986 (cell wall and cell processes), and MT3280 (unknown function)) have been associated with invasion or survival in the CNS but not in lung tissues in mice [22]. Especially *M. tuberculosis pknD* was associated with invasion of brain, but not lung epithelia in guinea pigs [23], as was confirmed by another study showing that *pknD* vaccination offered significant protection against bacterial dissemination to the brain in guinea pigs [24]. Similarly, in mice, clinical isolates from TBM patients disseminated extensively to cause meningitis, whereas *M. tuberculosis* H37Rv and clinical isolates from PTB patients did not [25]. In rabbits, production of phenolic glycolipid has been linked with the increased propensity of East-Asian/Beijing strains to cause TBM [26]. Finally, four *M. tuberculosis* genes were crucial for invading an artificial blood brain barrier in an in vitro model using primary human brain microvascular endothelial cells: *PE-PGRS18* (unknown function), Rv0987 (cell wall and cell processes), *grcC2* (intermediary metabolism and respiration), and *PPE29* (unknown function) [27].

Much less is known about the role of *M. tuberculosis* genotype in TBM in humans. Most studies have examined associations of *M. tuberculosis* lineage with disease phenotype. Compared to other lineages, strains belonging the East-Asian lineage were associated with extrapulmonary tuberculosis in one study [28], but not in another [29], while other studies found no association of *M. tuberculosis* lineage and disease localisation [30, 31]. Specifically looking at TBM, one study from Vietnam found the Euro-American lineage to be associated with PTB rather than TBM [32]. Only one study used whole genome sequencing to compare strains from TBM and PTB patients; large-scale and smaller genomic rearrangements, inversions, indels and single nucleotide polymorphisms (SNPs) in eight cerebrospinal fluid (CSF)-derived strains were not found in 69 comparison respiratory strains isolated from independent sputum samples [33]. In the current study, we used a much larger set of isolates and a novel approach to examine the effect of the *M. tuberculosis* genotype on the susceptibility to TBM. We compared *M. tuberculosis* genomes isolated from 216 PTB patients and 106 TBM patients from Indonesia, all HIV-negative, to detect homoplastic genetic variants associated with either PTB or TBM.

## Results

### Lineage distribution and phylogeny construction

*M. tuberculosis* isolates from established patient cohorts in Bandung, Indonesia were selected for whole genome sequencing. All available *M. tuberculosis* strains isolated from HIV-negative TBM patients and randomly selected strains from twice as many PTB patients from the same setting were included, one strain was selected per patient. Compared to the 216 PTB patients, the 106 TBM patients were from a similar ethnic background, but slightly younger, more often male, and more often previously treated for TB (Additional file 1: Table S1). Based on a 62-SNP barcode [34] 61.5% of the strains belonged to the Euro-American lineage (63.4% for PTB; 57.5% for TBM), 36% to the East-Asian lineage (33.3% for PTB; 41.5% for TBM), and 2.5% to the Indo-Oceanic lineage (3.2% for PTB; 0.9% for TBM). The lineage distribution did not differ significantly for strains isolated from TBM compared to PTB patients (Chi-square = 3.230; *p* = 0.199).

**Fig. 1:**
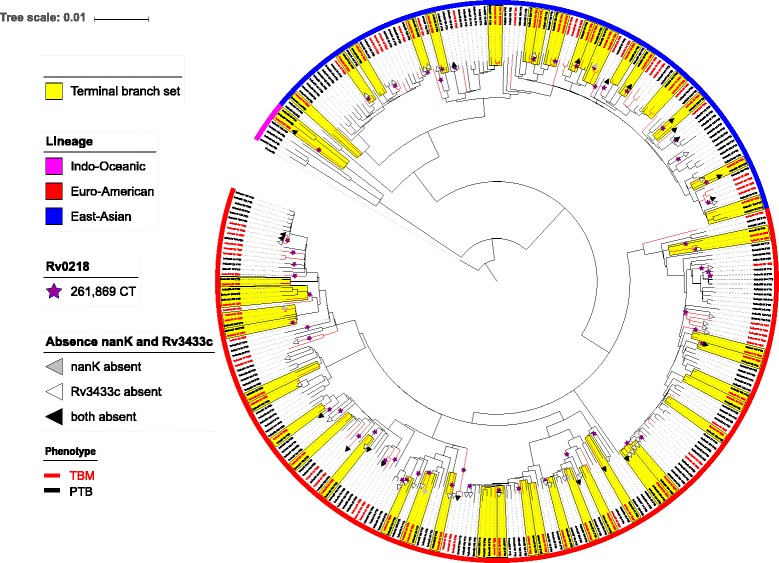
Phylogenetic tree of 322 *M. tuberculosis* strains isolated from TBM and PTB patients. Legend: The highlighted branches indicate the 108 strains in 47 terminal branch sets, together comprising the discovery set. The purple stars indicate the origin of the SNP in Rv0218 according to the ancestral reconstruction. The grey, white, and black triangles indicate the isolates in which *nanK* and/or Rv3433c are absent

A phylogenetic tree was constructed based on 28,544 variable common nucleotide positions among the 322 *M. tuberculosis* isolates. The phylogeny showed that the TBM phenotype was not restricted to a certain *M. tuberculosis* lineage; instead it arose many times independently (Fig. 1). In addition, the tree showed a high degree of strain heterogeneity. On average, two strains differed by about 1000 SNPs, and this pairwise distance did not differ among PTB and TBM strains (data not shown), indicating that there was equal genetic diversity within PTB and within TBM strains. In addition, the minimum pairwise genetic distance varied from 17 to 689 SNPs (data not shown), indicating that there was no clustering of strains (≤12 SNPs distance [35]). For TBM strains the minimum genetic distance ranged from 17 to 1785, and for PTB strains from 31 to 803 SNPs (data not shown).

### TB phenotype-associated genetic variations

**Fig. 2:**
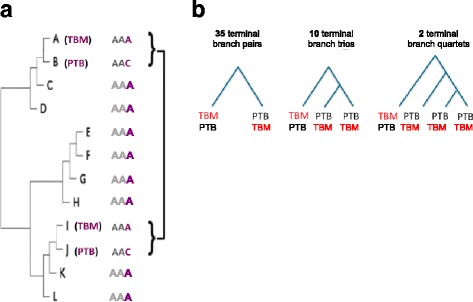
Homoplasy-based association analysis; detection of mutations occurring along disparate locations in the phylogenetic tree. Legend: (**a**) schematic example of a tree containing two terminal branch sets (TBSs) with a homoplastic SNP. **b** Schematic overview of the composition of the 47 terminal branch sets

Genome-wide association approaches for bacteria can broadly be categorised into allele counting and homoplasy counting methods [36]. Allele counting methods are based on the overrepresentation of an allele at the same site in cases relative to controls, introducing a risk of false-positive findings due to population stratification. Homoplasy counting on the other hand, counts repeated and independently emerging mutations that occur more often in branches of cases relative to controls. In the current study, we used a two-step approach: in the discovery phase, we used homoplasy counting by identifying terminal branch sets (TBSs) to maximize power to identify true associations with the TB disease phenotype, uncorrected for multiple tests. In the validation phase we examined associations identified in the discovery phase using allele counting with correction for multiple testing and for phylogenetic bias to distinguish true associations from false positives, and performed ancestral reconstruction to remove possible phylogenetic bias. To divide the genomes in a discovery and a validation set, we identified isolates in terminal branch pairs, trios and quartets (i.e. separated at a terminal or near terminal branch in the phylogenetic tree) with distinct phenotypes (Fig. 2). Genetic differences between isolates within a TBS provide the strongest, homoplasy-corrected possible association with the phenotype.

The phylogenetic tree revealed a total of 47 TBSs containing 108 paired strains: 54 TBM and 54 PTB strains that make up the discovery set. The merged SNP lists consisted of 6488 variable positions with the corresponding nucleotides for the 108 strains (Additional file 2). Using the homoplasy-based association analysis, we found individual nucleotide positions, genes, or pathways where TB disease phenotype-associated mutations repeatedly occur in different branches of the phylogenetic tree. These included 9 SNPs, 5 genes, and 1 pathway (Table 1).

**Table 1:** Significant SNPs, genes, and pathways identified by homoplasy counting

		Discovery dataset (*n* = 108)			Validation dataset (*n* = 214)		
SNP-level		Strains with SNP (N)			Strains with SNP (N)		
Gene (Rv-number)	Nucleotide change	TBM (*n* = 54)	PTB (*n* = 54)	*P*-value	TBM (*n* = 52)	PTB (*n* = 162)	*P*-value^
*Unnamed* (Rv0218)	261,869CT	25	10	0.002	21	24	**0.001**
*PPE54* (Rv3343c)	3736,628TG	22	35	0.008	27	87	0.472
*PEPGRS19* (Rv1067c)	1,190,093AC	2	11	0.01	5	18	0.487
*PEPGRS44* (Rv2591)	2922,848AT	0	6	0.01	2	7	0.625
*PPE3* (Rv0280)	340,372TC	2	10	0.025	4	13	0.589
*PEPGRS9* (Rv0746)	836,272AG	7	1	0.029	3	9	0.603
*PEPGRS26* (Rv1441c)	16,18,978TC	0	5	0.032	2	3	0.369
*Unnamed* (Rv0064)	713,36GC	24	34	0.034	29	98	0.355
*PEPGRS18* (Rv0980c)	1095,644CT	0	4	0.044	2	2	0.238
Gene-level		SNPs in gene (N)			SNPs in gene (N)		
Gene (Rv number)		TBM	PTB	*P*-value	TBM	PTB	*P*-value
*PEPGRS19* (Rv1067c)		2	13	0.004	5	22	0.318
*Unnamed* (Rv0218)		27	11	0.007	21	32	**0.001**
*PEPGRS26* (Rv1441c)		0	5	0.031	2	4	0.491
*glmS* (Rv3436c)		0	5	0.031	0	0	n.a.
*Unnamed* (Rv3740c)		0	4	0.05	3	0	0.011
Pathway-level		Genes with mutation (N)			Genes with mutation (N)		
Pathway name		TBM	PTB	***P-value***	TBM	PTB	*P*-value
Ethylbenzene degradation		68	54	0.032	65	224	0.071

**NOTE**. *P*-values are based on permutation analysis; bold *p*-values indicate validated, Bonferroni-corrected significant enrichment

^ *P*-value thresholds in the validation set were Bonferroni-corrected for multiple testing by dividing them by the number of top hits in the discovery set: SNP-level: *p* < 0.05/9; gene-level: *p* < 0.05/5; pathway-level: *p* < 0.05/1

We used the remaining 214 (52 TBM and 162 PTB) isolates not belonging to any of the TBSs (validation set) to verify these results. The discovery set showed a total of 6488 different non-synonymous SNPs involving 6483 dimorphic sites and 5 trimorphic sites across 2778 genes; the validation set a total of 12,211 different non-synonymous SNPs involving 12,185 dimorphic sites and 26 trimorphic sites across 3359 genes (Additional file 3). There was an overlap of 2694 non-synonymous SNPs and 1564 affected genes. Out of 9 SNPs significantly associated with either TBM or PTB in the discovery set, one was confirmed in the validation set; the mutation in Rv0218. Similarly, out of 5 genes harbouring genetic variation associated with the TB phenotype, Rv0218 was validated in the validation set (Table 1, Additional file 4: Figure S1 and Additional file 5: Figure S2). The pathway (ethylbenzene degradation) identified in the discovery set was not confirmed in the validation set.

To correct for potential phylogenetic bias in the validation set, we reconstructed the ancestral state for the SNP in Rv0218 and compared the ratio of TBM vs. PTB isolates after the occurrence of this particular SNP with the ratio of TBM vs. PTB prior to the occurrence of this SNP in the validation set (Fig. 1 and Additional file 6: Figure S3). Three branches with back mutations (2 TBM, 1 PTB branch) were excluded from the analysis. Among the 33 nodes / leaves where the SNP occurred, the average unweighted proportion of TBM isolates among the child branches was 44.7%; among 166 isolates in the validation set not harbouring this SNP, 29 (17.5%) were from TBM patients (Additional file 7: Table S2). The Z-score for the difference between proportions was − 3.83 (*p* < 0.001).

### *De novo* genome assembly

Next, we used a reference-free *de novo* genome assembly approach to examine associations between presence/absence of genes and the TB phenotype, and to study sequences not present in the reference genome. The list of annotated coding sequences for the 108 assembled genomes in the discovery set contained 3032 distinct genes that were present in at least one, but not all of the strains (Additional file 8). The permutation analysis revealed six genes that were significantly associated with the TB phenotype. Two of these genes, Rv3433c and *nanK*, were validated in the validation set: absence of these genes was associated with TBM (Fig. 1, Table 2, Additional file 9). Together with Rv0218 they bring the total number of genes associated with the TB phenotype to three.

**Table 2:** Significant genes identified by the *de novo* genome assembly analysis

	Discovery dataset (*n* = 108)			Validation dataset (*n* = 214)		
	Strains with CDS present (N)			Strains with CDS present (N)		
Coding sequence	TBM (*n* = 54)	PTB (*n* = 54)	*P*-value	TBM (*n* = 52)	PTB (*n* = 162)	*P*-value^
Bifunctional NAD(P)H-hydrate repair enzyme Nnr (Rv3433c)	36	47	0.011	34	144	**0.002**
TMAO/DMSO reductase	46	53	0.016	49	152	0.592
Antitoxin/MT2731	33	44	0.017	37	130	0.206
NPCBM-associated, NEW3 domain of alpha-galactosidase	15	6	0.022	14	19	0.063
Oxidoreductase molybdopterin binding domain protein	11	3	0.028	7	13	0.197
N-acetylmannosamine kinase (*nanK*)	44	51	0.036	44	157	**0.001**

**NOTE**. *P*-values are based on permutation analysis; bold *p*-values indicate validated significant enrichment

^ *P*-value thresholds in the validation set were Bonferroni-corrected for multiple testing by dividing them by the number of top hits in the discovery set: *p* < 0.05/6

### Effect of detected SNPs on protein function and predicted function of phenotype-associated genes

We used published algorithms to predict the effects of identified mutations on protein structure and function. The SNP in Rv0218, a protein predicted to have transmembrane helices, likely leads to a decrease of stability of the protein (Additional file 10: Table S3). For Rv3433c, and *nanK* no transmembrane helices or signalling peptides were predicted.

## Discussion

To determine whether *M. tuberculosis* genetic variation is associated with the TB disease phenotype, we compared *M. tuberculosis* whole genome sequences from 216 PTB and 106 TBM patients and searched for homoplastic mutations. We identified three genes in *M. tuberculosis* (Rv0218, Rv3433c, and *nanK*) to be associated with either TBM or PTB. Previous experimental studies have assessed the importance of Rv0218. This secretome gene encodes for a protein with multiple predicted transmembrane regions and a C-terminal molybdopterin binding domain that is often found in oxidoreductases and was shown to be essential for *M. tuberculosis* in vivo growth in C57BL/6 J mouse spleen [37]. The SNP in Rv0218 is predicted to decrease the stability of the respective protein. Secretome genes potentially influence pathogen recognition and host-pathogen interaction [38]. If mutations in these genes alter the appearance of the *M. tuberculosis* surface, this could provide a mechanism by which *M. tuberculosis* could evade the immune response and enable dissemination to extrapulmonary sites. Secretome genes are more likely to contain false-positive associations as they are under selective pressure from the immune system and phages [39]. How Rv3433c and *nanK* could be related to the TB phenotype is not obvious,although functions have been predicted based on homology detection.

To our knowledge, this is only the second attempt to relate *M. tuberculosis* genetic variation to the TB disease phenotype in humans on a genome-wide scale. The other study, by Saw et al. showed large-scale rearrangements, short translocations, inversions, indels and SNPs in eight strains cultured from CSF [33]. Non-synonymous SNPs in eight genes (*embR*, *lppD*, *PE-PGRS10*, *PE-PGRS19*, *PE-PGRS21*, *PE-PGRS49*, *PPE58*, and Rv0278c) were found in at least four of the eight CSF-derived strains, and in none of 69 strains isolated from sputum [33]. We did not confirm this in our set of isolates, although *PE-PGRS19* was associated with the TB phenotype in the discovery set. Moreover, we used a two-step approach, based on homoplasy counting as well as allele counting with a correction for phylogenetic bias to find mutations associated with the TB phenotype, and we performed ancestral reconstruction for the most discriminative SNP. Unlike a previous study from Vietnam [32], we found no association between *M. tuberculosis* lineage and TBM. This is no surprise given the genetic diversity even within *M. tuberculosis* lineages [9], and the observed pattern of TBM isolates scattered across the phylogenetic tree.

In concordance with previous findings [9], we found considerable genetic diversity in *M. tuberculosis* in the current study. Two isolates differed on average by 1000 SNPs, and this did not differ among PTB isolates and among TBM isolates. In addition, we did not observe any clustering, defined as two isolates differing by 12 SNPs or less [35]. The lack of clustering is probably a result of the low sampling fraction in this urban setting with thousands of incident TB cases each year.

Theoretically, two scenarios could explain the role of *M. tuberculosis* genetic variation in the development of TBM after infection with *M. tuberculosis*. First, upon infection the *M. tuberculosis* strain may carry certain mutations associated with dissemination and penetration of the blood-brain barrier. Second, a subpopulation of bacteria in the lungs of a PTB patient may develop such mutations, though it was recently shown for bacterial meningitis caused by *S. pneumonia* or *N. meningitides* that there is no evidence for differential selection between blood and CSF, and that any mutations between these two niches is likely due to mutation hotspots or forms of diversifying selection common to both niches [40]. However, similar to the findings of Saw et al. [33], the genetic variants that we found to be associated with the TB disease phenotype were not exclusive for TBM or PTB, nor were they consistently present in all TBM or PTB strains. Therefore it seems that genetic variants may be part of a complex, multifactorial process leading to this devastating manifestation of TB, in which the human genotype or phenotype equally plays an important role [32, 41].

This is the second, and by far largest study using whole genome sequencing to link *M. tuberculosis* genotype to TBM. In this large cohort of well-characterised patients we studied strains from HIV-negative, adult patients to control for the two most important known risk factors for TBM. In addition, both patient groups were similar with regard to gender, ethnicity, and previous episodes of TB. The *de novo* assembly adds to the strengths of this study because it enabled us to examine regions of the genome that do not map to the reference genome, allowing the investigation of associations between genetic variation in these genomic regions and the TB disease phenotype. The homoplasy-based association analysis has proven to be a successful method to detect *M. tuberculosis* loci associated with a certain phenotype (e.g. transmissible vs. non-transmissible, drug-resistant vs. sensitive) [42, 43]. The major advantage is that false-positive associations due to genetic relatedness of strains with the same phenotype (i.e. ‘phylogenetic bias’) are filtered out, thereby increasing statistical power to find true associations. In addition, the ancestral reconstruction in the validation step ruled out the possibility that the significant association for the SNP in Rv0218 was due to population structure.

The current study has several limitations. Firstly, we only focused on mutations in coding regions of the genome, as they are more likely to have functional consequences, but mutations in non-coding regions could also affect function, for instance by transcriptional and translational regulation of protein-coding sequences [44]. Secondly, the large number of genetic variants increases the risk of finding false-positive associations, although homoplasy counting enabled us to filter out many of these false-positives. We did not correct for multiple testing in the discovery set, but we used a validation set where we did correct for multiple testing for confirmation. Lastly, whether bacteria developed TBM-associated mutations before or after infecting a patient remains unclear. One way to investigate this is to compare the genomes of strains isolated from sputum and CSF from the same patient. Unfortunately we did not have the availability of paired isolates. Most TBM patients were too ill to expectorate sputum.

## Conclusions

We present evidence from a homoplasy-based association analysis that three *M. tuberculosis* genes, including Rv0218, a cell wall-associated and/or secretome gene, are associated with the TB disease phenotype. These findings serve as an important step forward in the quest for an improved understanding of the mycobacterial determinants of TB tissue tropism. Functional validation studies are warranted to further explore the effect of mutations in these genes on protein function.

## Methods

### Patients and isolates

We used *M. tuberculosis* isolates from two established cohorts of Indonesian patients with confirmed TB. The first group consisted of adult patients (≥15 years old) with TBM admitted at Hasan Sadikin Hospital between 2006 and 2013, with *M. tuberculosis* cultured from CSF. The second group was randomly selected from a cohort of culture-positive HIV-negative PTB patients (age ≥ 15 years) from the same setting recruited between 2012 and 2015. All patients were tested for HIV, and those who were HIV-positive were excluded.

### Sequencing, alignment, and variant calling

Mycobacterial DNA was extracted from cultures using cetyl trimethylammonium bromide (CTAB) or using UltraClean® Microbial DNA Isolation Kit (MO BIO Laboratories). A single isolate from each patient was selected for sequencing. *M. tuberculosis* DNA was sequenced on an Illumina HiSeq 2000 instrument using 2 × 100 bp paired-end reads at the Beijing Genome Institute in Hong Kong. After sequencing, the raw FASTQ sequence reads were filtered, including removing of adapter sequences, contamination, and low quality reads which have more than 10% N base calls, or where more than 40% of the bases have a quality score ≤ 4. Quality control statistics are shown in Additional file 11: Table S4. Five TBM strains and four PTB strains were contaminated, based on a low GC-content, and were excluded from further analyses. Sequencing coverage was determined using the FASTQC quality control tool version 0.10.1. The proportion of bases sequenced with a sequencing error rate of 1% or less per base ranged from 93% to 97% per genome. The average coverage depth for the remaining 322 sequenced strains was 121.1, and the average percentage of bases covered by at least one read was 98.9%.

The sequence reads were aligned to reference strain *M. tuberculosis* H37Rv, accession number NC_000962.3, and variants were called using Breseq software, version 0.27.1 [45] using a minimum threshold of 30× coverage. Mutations with low-quality evidence (i.e. possible mixed read alignment) were not included. The Breseq variant call output was converted to a tab-separated file for each sequence using customized Python and R scripts that are available upon request.

### Phylogeny construction

A phylogeny was constructed to determine evolutionary relationships of the isolates. We extracted all 29,199 variable positions across the 322 *M. tuberculosis* sequences and concatenated them into a single alignment. Solely for the purpose of creating the phylogenetic tree, SNPs occurring in PE/PPE genes and genes related to mobile elements (genes listed in Additional file 12: Table S5) were excluded to avoid any concern about inaccuracies in the read alignment in these parts of the genome. In addition, SNPs in an additional 40 genes previously associated with drug resistance [46] were removed to exclude the possibility that homoplasy of drug resistance mutations would significantly affect the phylogeny [47]. After applying these filters to the initial set of 29,199 SNPs, the 28,544 remaining SNPs were used to construct the phylogenetic tree using PhyML, version 3.0 [48] using the HKY85 model with four categories for the gamma distribution, and using a hundred bootstraps.

To determine the lineage distribution of the strains and to evaluate whether an association exists between *M. tuberculosis* lineage and TB disease phenotype, we determined the lineage for each of the 322 strains using a 62-SNP barcode [34]. The resulting classification in the main *M. tuberculosis* lineages also served as a quality check for the generated Maximum Likelihood (ML)-phylogenetic tree, as it enabled us to validate that isolates belonging to the same lineage clustered together in the tree. A Chi-square test was used to statistically test the association between *M. tuberculosis* lineage and TB disease phenotype.

### Homoplasy-based association test to identify associations between M. Tuberculosis genotype and TB disease phenotype

We used a two-step approach: in the discovery step we aimed to maximize power by homoplasy counting, without correction for multiple testing. In the subsequent validation step, aimed to distinguish true associations from false positives, we used allele counting with multiple testing correction, and performed ancestral reconstruction to remove possible phylogenetic bias.

To divide the genomes in a discovery and a validation set, we identified isolates in terminal branch pairs, trios and quartets (i.e. separated at a terminal or near terminal branch in the phylogenetic tree) with distinct phenotypes (Fig. 2). These terminal branch sets (TBSs) together formed the discovery set. These provide the strongest, homoplasy-corrected possible association with the phenotype. We used the remaining genomes to validate the association. For all isolates in the TBSs, we listed the non-synonymous SNPs to create a table with all variable positions in rows, the paired isolates in columns, and the corresponding nucleotide in the cells (Additional file 2). For every SNP, an enrichment score was calculated using the following formula:

$$ \log \left(\frac{\left( number\ of\  TBM\  isolates\ with\  SNP/ total\ number\ of\  TBM\  isolates\right)+0.001}{\left( number\ of\  PTB\  isolates\ with\  SNP/ total\ number\ of\  PTB\  isolates\right)+0.001}\right) $$

A permutation *p*-value for each SNP was calculated by randomising the phenotypes over the isolates 1000 times.

In parallel we grouped SNPs per gene, using the same empirical randomisation strategy to assess association, adjusted for gene length:

$$ \mathit{\log}\left(\frac{\left(\left( number\ of\ SNPs\ in\ gene\ in\  TBM\  isolates/ gene\ length\right)/ total\ number\ of\  TBM\  isolates\right)+0.001}{\left(\left( number\ of\ SNPs\ in\ gene\ in\  PTB\  isolates/ gene\ length\right)/ total\ number\ of\  PTB\  isolates\right)+0.001}\right) $$

Similarly, we grouped genes with ≥1 SNP per *M. tuberculosis* pathway according to PATRIC [49], and calculated association using the aforementioned permutation analysis, adjusted for the number of genes in a particular pathway:

$$ \mathit{\log}\left(\frac{\left(\left( number\ of\ pathway\ genes\ with\ge 1\  SNP\  in\  TBM\  isolates/ unique\ gene\ count\right)/ total\ number\ of\  TBM\  isolates\right)+0.001}{\left(\left( number\ of\ pathway\ genes\ with\ge 1 SNP\  in\  PTB\  isolates/ unique\ gene\ count\right)/ total\ number\ of\  PTB\  isolates\right)+0.001}\right) $$

Significance of associations was determined by calculating a permutation *p*-value through randomization of the phenotypes over the isolates 1000 times. All calculations were performed with customized Perl scripts that are available upon request.

We used the set of 214 strains that were not in TBSs to validate candidate SNPs, genes, and pathways identified in the discovery set, using the same permutation test as described above for the discovery set. We used a *p*-value threshold of 0.05 for the discovery set. The *p*-value thresholds in the validation set were Bonferroni-corrected for multiple testing by dividing them by the number of significant (candidate) hits in the discovery set. To correct for potential phylogenetic bias in the validation set, we performed ancestral reconstruction for validated TB phenotype-associated SNPs using FASTML [50] with default parameters, and compared the proportion of TBM vs. PTB isolates prior to (i.e. older than) and after (i.e. younger than) the occurrence of the SNP in the validation set. For each node / leave where the SNP occurred, we calculated the proportion of TBM isolates among the child branches and we calculated the (unweighted) average over all of these nodes and leaves to determine the proportion of TBM isolates after the SNP (Additional file 7: Table S2). This way, every independent occurrence of the SNP contributes equally to the analysis, regardless of the number of child branches after the SNP, thus correcting for phylogenetic bias. The significance of the difference in proportion was determined by calculating the Z-score for 2 population proportions with accompanying p-value.

PE/PPE genes, a major challenge in the analysis of *M. tuberculosis* whole genome sequences due to the repetitive nature of these sequences, were included in the analysis. TB phenotype-associated SNPs in PE/PPE genes were manually examined to confirm that they did not fall within a repetitive region (for an example please see Additional file 13: Figure S4).

### *De novo* genome assembly

Sequence reads were *de novo* assembled using SPAdes, version 3.6.1 [51] with default parameters. All assemblies were evaluated, focussing on genome size, N50 length, number of contigs and scaffolds, and GC-content. The assembled genomes were annotated using Prokka, version 1.11 [52] with default parameters. Completeness and contamination of assemblies were determined with CheckM version 1.0.5 [53]. The assembly statistics are shown in Additional file 14. Presence or absence of annotated genes was determined for the 108 assembled genomes in the discovery set. An enrichment score per gene was calculated based on the frequency of occurrence in TBM vs. PTB strains using the following formula:

$$ \log \left(\frac{\left( number\ of\  TBM\  isolates\ with\ gene\ present/ total\ number\ of\  TBM\  isolates\right)+0.001}{\left( number\ of\  PTB\  isolates\ with\ gene\ present/ total\ number\ of\  PTB\  isolates\right)+0.001}\right) $$

Statistical significance was again determined based on permutation by randomizing the phenotypes over the isolates 1000 times. We repeated this permutation analysis for the 214 genomes comprising the validation set, using Bonferroni-adjusted *p*-value thresholds. For the genes with a validated, significant enrichment for TBM or PTB, we confirmed their absence in the respective genomes by mapping the raw sequencing reads for these genomes back to the H37Rv reference sequence of the gene (Additional file 15: Figure S5), and visualized this with integrative genomics viewer (IGV), version 2.3.32 [54].

### Prediction of mutation effects

We used two algorithms to predict the effect of the mutations on protein structure and function. I-Mutant version 2.0, which predicts the protein stability change upon single site mutation (http://folding.biofold.org/i-mutant/i-mutant2.0.html) [55] and PolyPhen-2, which predicts the possible impact of an amino acid substitution on the structure and function of a protein (http://genetics.bwh.harvard.edu/pph2/) [56] to predict the impact of the validated SNPs on protein structure and function. In addition, we used TartgetP (http://www.cbs.dtu.dk/services/TargetP/) [57] to predict the subcellular location of the proteins encoded by the validated genes, and TMHMM (http://www.cbs.dtu.dk/services/TMHMM/) [58] to predict transmembrane helices in these proteins.

## Additional files

Additional file 1: Table S1. Description of baseline characteristics for PTB and TBM patients. IQR, interquartile range; SD, standard deviation. Data were missing for history of TB treatment (TBM, *n* = 7; PTB, *n* = 1); ethnicity (TBM, *n* = 58; PTB = 2). (DOCX 58 kb)

Additional file 2: Merged SNP lists consisting of variable positions for the 108 strains in the discovery set. File showing all common non-synonymous SNPs in the 108 isolates in the discovery set, including their *p*-value for association with the TB disease phenotype. (XLSX 2729 kb)

Additional file 3: Merged SNP lists consisting of variable positions for the 214 strains in the validation set. File showing all common non-synonymous SNPs in the 214 isolates in the validation set, including their *p*-value for association with the TB disease phenotype. (XLSX 8758 kb)

Additional file 4: Figure S1. *P*-values of association between SNPs and TB disease phenotype in the discovery and validation sets. Scatterplot showing the *p*-values of the SNPs found in the discovery and the validation set. *P*-values in the discovery set are shown on the x-axis; p-values in the validation set are shown on the y-axis. Gene names are shown for SNPs significant in both the discovery and validation set. (DOCX 82 kb)

Additional file 5: Figure S2. *P*-values of association between genes and TB disease phenotype in the discovery and validation sets. Scatterplot showing the *p*-values of the genes found in the discovery and the validation set. *P*-values in the discovery set are shown on the x-axis; *p*-values in the validation set are shown on the y-axis. Names are shown for genes significant in both the discovery and validation set. (DOCX 66 kb)

Additional file 6: Figure S3. Phylogenetic tree of 322 *M. tuberculosis* strains isolated from TBM and PTB patients. The highlighted branches indicate the 108 strains in 47 terminal branch sets, together comprising the discovery set. The purple stars indicate the origin of the SNP in Rv0218 according to the ancestral reconstruction. The nucleotide for SNP position 261,869 is indicated next to the leaf labels. (PDF 60 kb)

Additional file 7: Table S2. Ancestral reconstruction of SNP 261869TC in Rv0218. Listed are the internal nodes and leaves where the SNP in Rv0218 occurred according to the ancestral reconstruction of the SNP. (DOCX 72 kb)

Additional file 8: Annotated coding sequences present in the 108 *de novo* assembled genomes in the discovery set. Annotated coding sequences present in at least one, but not in all of the 108 *de novo* assembled genomes in the discovery set. (XLSX 1286 kb)

Additional file 9: Annotated coding sequences present in the 214 *de novo* assembled genomes in the validation set. Annotated coding sequences present in at least one, but not in all of the 214 *de novo* assembled genomes in the validation set. (XLSX 1309 kb)

Additional file 10: Table S3. Protein prediction for genomic sites associated with the TB disease phenotype. NA: No homologs of Rv0192 were found therefore protein prediction was not possible. ^#^ I-mutant predicts free energy changes of protein stability upon a point mutation under different conditions. ^&^ PolyPhen predicts the possible impact of an amino acid substitution on the structure and function of a human protein using straightforward physical and comparative considerations. ^^^ TargetP predicts the subcellular location of proteins based on the predicted presence of any N-terminal signal peptides. ^*^ TMHMM predicts transmembrane helices in proteins. (DOCX 57 kb)

Additional file 11: Table S4. Description of sequencing quality control parameters and statistics. Displayed are different measures of sequencing quality, used for the sequencing quality control check. (DOCX 208 kb)

Additional file 12: Table S5. PE / PPE genes and drug resistance genes excluded for the phylogeny construction. Listed are the genes that were excluded from the multiple alignment used to create the phylogenetic tree. (DOCX 158 kb)

Additional file 13: Figure S4. Diagram demonstrating breseq calling a SNP in the *PE-PGRS1* gene. Displayed are 60 Illumina sequencing reads mapping to the H37Rv reference genome (shown at the top and bottom). Visual inspection of the SNP confirms that it does not occur in a region containing uniformly lower base quality scores. (DOCX 1204 kb)

Additional file 14: *De novo* genome assembly statistics. File listing different measures used to check the quality of the *de novo* genome assemblies. (XLS 73 kb)

Additional file 15: Figure S5. Read alignment demonstrating the absence of *nanK*. Displayed is the alignment of the raw sequencing reads against the H37Rv *nanK* gene. No reads are mapping to this gene, showing that it is absent in the sequenced genome. (DOCX 54 kb)
