# Supplementary material for: Large-scale genomic analysis shows association between homoplastic genetic variation in Mycobacterium tuberculosis genes and meningeal or pulmonary tuberculosis
Source: BMC Genomics. 2018 Feb 5;19:122. doi: 10.1186/s12864-018-4498-z (PMC5800017; doi:10.1186/s12864-018-4498-z)
Supplement: Supplementary file 1 — Description of baseline characteristics for PTB and TBM patients. IQR, interquartile range; SD, standard deviation. Data were missing for history of TB treatment (TBM, n = 7; PTB, n = 1); ethnicity (TBM, n = 58; PTB = 2). (DOCX 58 kb) [file 12864_2018_4498_MOESM1_ESM.docx]

**Additional Table 1**. Description of baseline characteristics for PTB and TBM patients.

| **Characteristic** | **TBM patients (N=106)** | **PTB patients (N=216)** |
| --- | --- | --- |
| Male gender – N (%) | 58 (54.7) | 111 (51.4) |
| Age (yrs) – mean (SD) | 28.0 (8.9) | 39.1 (14.8) |
| Ethnicity – N (%)  Sundanese  Javanese  Sumatra  Other | 45 (93.7)  3 (6.3)  0 (0)  0 (0) | 187 (87.4)  21 (9.8)  3 (1.4)  3 (1.4) |
| Years after admission – median (IQR) | 6 (4-8) | 1 (1-2) |
| History of TB treatment - N (%) | 13 (13.1) | 37 (17.2) |

IQR, interquartile range; SD, standard deviation.

* Data were missing for history of TB treatment (TBM, n=7; PTB, n=1); ethnicity (TBM, n=58; PTB = 2).
