# Supplementary figures and images for: Large-scale genomic analysis shows association between homoplastic genetic variation in Mycobacterium tuberculosis genes and meningeal or pulmonary tuberculosis
Source: BMC Genomics. 2018 Feb 5;19:122. doi: 10.1186/s12864-018-4498-z (PMC5800017; doi:10.1186/s12864-018-4498-z)

Terminal branch set

Lineage

- Indo-Oceanic
- Euro-American
- East-Asian

Rv0218

★ 261869CT

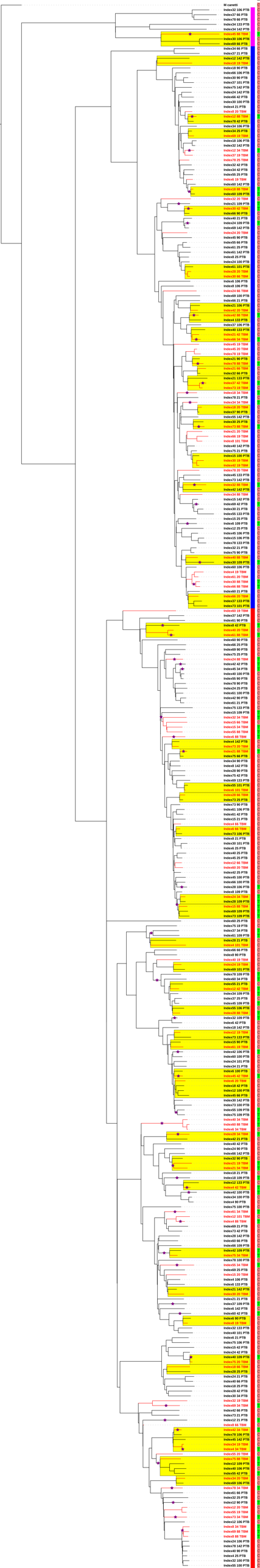

Supplement: Supplementary file 6 — Phylogenetic tree of 322 M. tuberculosis strains isolated from TBM and PTB patients. The highlighted branches indicate the 108 strains in 47 terminal branch sets, together comprising the discovery set. The purple stars indicate the origin of the SNP in Rv0218 according to the ancestral reconstruction. The nucleotide for SNP position 261,869 is indicated next to the leaf labels. (PDF 60 kb) [file 12864_2018_4498_MOESM6_ESM.pdf]

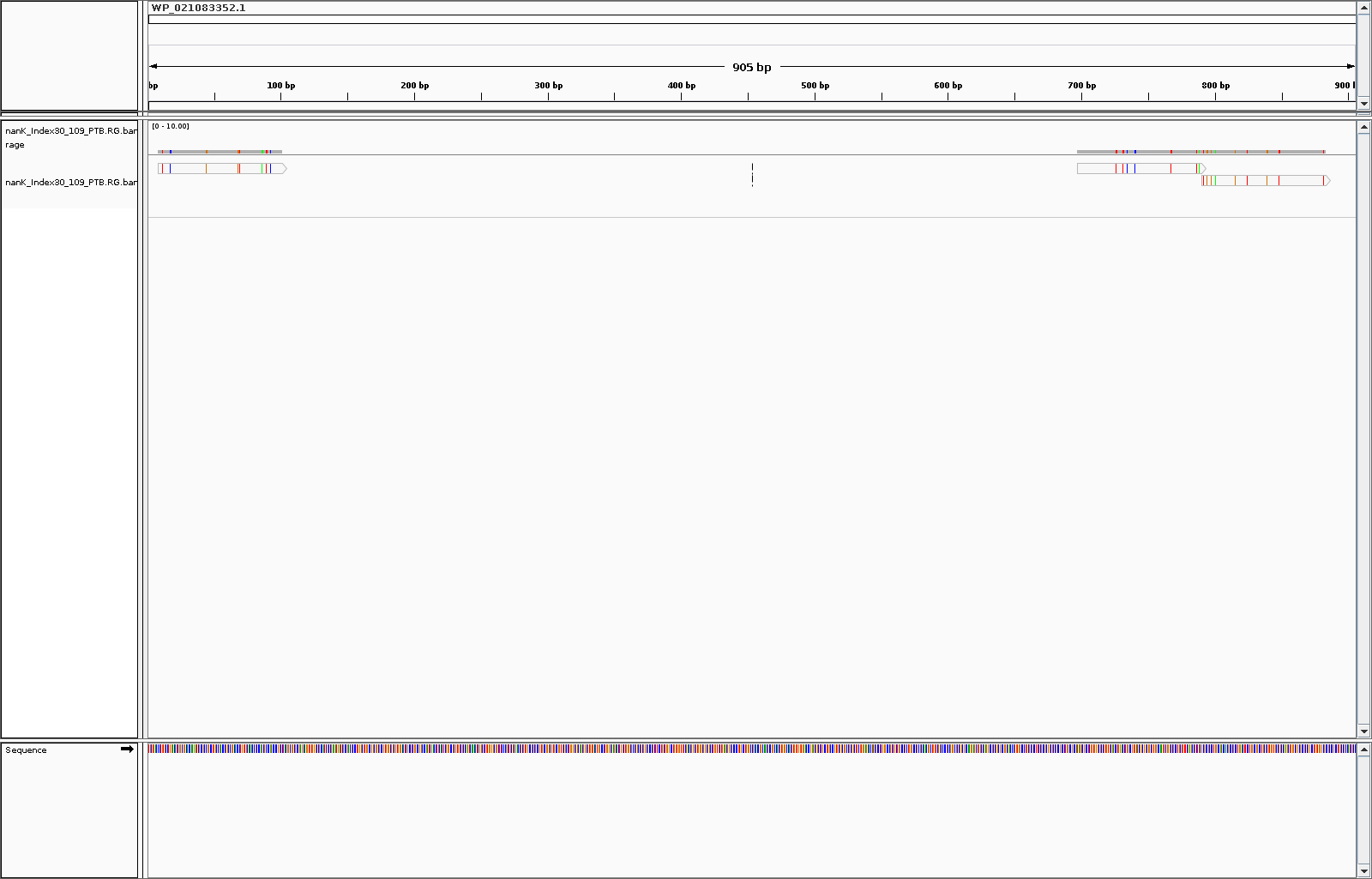

Supplement: Supplementary file 15 — Read alignment demonstrating the absence of nanK. Displayed is the alignment of the raw sequencing reads against the H37Rv nanK gene. No reads are mapping to this gene, showing that it is absent in the sequenced genome. (DOCX 54 kb) [file 12864_2018_4498_MOESM15_ESM.docx]
