# Supplementary material for: Large-scale genomic analysis shows association between homoplastic genetic variation in Mycobacterium tuberculosis genes and meningeal or pulmonary tuberculosis
Source: BMC Genomics. 2018 Feb 5;19:122. doi: 10.1186/s12864-018-4498-z (PMC5800017; doi:10.1186/s12864-018-4498-z)
Supplement: Supplementary file 7 — Ancestral reconstruction of SNP 261869TC in Rv0218. Listed are the internal nodes and leaves where the SNP in Rv0218 occurred according to the ancestral reconstruction of the SNP. (DOCX 72 kb) [file 12864_2018_4498_MOESM7_ESM.docx]

| **Node/leave where SNP occurred** | **Number of child branches** | **Number of TBM branches among children** | **Percentage TBM branches among children** |
| --- | --- | --- | --- |
| Index12_34_TBM | 1 | 1 | 100 |
| Index21_109_PTB | 1 | 0 | 0 |
| Index24_109_PTB | 1 | 0 | 0 |
| Index18_34_TBM | 1 | 1 | 100 |
| Index34_34_TBM | 1 | 1 | 100 |
| Index69_42_PTB | 1 | 0 | 0 |
| Index6_109_PTB | 1 | 0 | 0 |
| Index30_88_TBM\|Index66_88_TBM | 2 | 2 | 100 |
| Index24_88_TBM | 1 | 1 | 100 |
| Index42_42_PTB | 1 | 0 | 0 |
| Index45_34_PTB | 1 | 0 | 0 |
| Index15_109_PTB\|Index32_34_TBM\|Index15_66_TBM\|  Index15_34_TBM\|Index55_88_TBM | 4 | 3 | 75 |
| Index6_88_TBM | 1 | 1 | 100 |
| Index28_106_PTB | 1 | 0 | 0 |
| Index37_34_PTB\|Index61_109_PTB | 2 | 0 | 0 |
| Index78_109_PTB\|Index60_34_PTB\|Index34_109_PTB\|  Index37_25_PTB\|Index45_109_PTB | 4 | 0 | 0 |
| Index32_109_PTB | 1 | 0 | 0 |
| Index42_106_PTB | 1 | 0 | 0 |
| Index55_109_PTB\|Index75_109_PTB | 2 | 0 | 0 |
| Index40_34_TBM\|Index60_88_TBM\|Index6_34_TBM | 2 | 2 | 100 |
| Index18_109_PTB | 1 | 0 | 0 |
| Index42_100_PTB | 1 | 0 | 0 |
| Index61_34_TBM | 1 | 1 | 100 |
| Index4_88_TBM | 1 | 1 | 100 |
| Index55_34_TBM | 1 | 1 | 100 |
| Index37_109_PTB | 1 | 0 | 0 |
| Index60_42_PTB | 1 | 0 | 0 |
| Index69_34_TBM | 1 | 1 | 100 |
| Index12_21_PTB | 1 | 0 | 0 |
| Index78_34_TBM | 1 | 1 | 100 |
| Index12_90_PTB | 1 | 0 | 0 |
| Index73_34_TBM | 1 | 1 | 100 |
| Index8_34_TBM\|Index69_88_TBM\|Index8_88_TBM | 3 | 3 | 100 |
| **Sum** | 45 | 21 | 1475 |
| **Average** | 1,36 | 0,64 | 44,70 |

**Additional Table 2.** Ancestral reconstruction of SNP 261869TC in Rv0218.
