# Supplementary material for: Large-scale genomic analysis shows association between homoplastic genetic variation in Mycobacterium tuberculosis genes and meningeal or pulmonary tuberculosis
Source: BMC Genomics. 2018 Feb 5;19:122. doi: 10.1186/s12864-018-4498-z (PMC5800017; doi:10.1186/s12864-018-4498-z)
Supplement: Supplementary file 10 — Protein prediction for genomic sites associated with the TB disease phenotype. NA: No homologs of Rv0192 were found therefore protein prediction was not possible. # I-mutant predicts free energy changes of protein stability upon a point mutation under different conditions. & PolyPhen predicts the possible impact of an amino acid substitution on the structure and function of a human protein using straightforward physical and comparative considerations. ^ TargetP predicts the subcellular location of proteins based on the predicted presence of any N-terminal signal peptides. * TMHMM predicts transmembrane helices in proteins. (DOCX 57 kb) [file 12864_2018_4498_MOESM10_ESM.docx]

**Additional Table 3.** Protein prediction for genomic sites associated with the TB disease phenotype

|  |  |  |  | Protein Prediction | | Predicted localization | Predicted number of transmembrane helices |
| --- | --- | --- | --- | --- | --- | --- | --- |
|  | Genomic position | Nucleotide change | Amino acid change | I-Mutant ^#^ | PolyPhen ^&^ | TargetP ^^^ | TMHMM ^*^ |
| **SNP-level** | **Rv0218** |  |  |  |  |  |  |
|  | 261,869 | C=>T | R316C | Decrease of Stability | Benign | Other | 5 |
| **Gene-level** | **Rv3433c** |  |  |  |  | Other | 0 |
|  | ***nanK*** |  |  |  |  | Other | 0 |

^#^ I-mutant predicts free energy changes of protein stability upon a point mutation under different conditions.

^&^ PolyPhen predicts the possible impact of an amino acid substitution on the structure and function of a human protein using straightforward physical and comparative considerations.

^^^ TargetP predicts the subcellular location of proteins based on the predicted presence of any N-terminal signal peptides.

^*^ TMHMM predicts transmembrane helices in proteins.
