# Supplementary material for: Large-scale genomic analysis shows association between homoplastic genetic variation in Mycobacterium tuberculosis genes and meningeal or pulmonary tuberculosis
Source: BMC Genomics. 2018 Feb 5;19:122. doi: 10.1186/s12864-018-4498-z (PMC5800017; doi:10.1186/s12864-018-4498-z)
Supplement: Supplementary file 11 — Description of sequencing quality control parameters and statistics. Displayed are different measures of sequencing quality, used for the sequencing quality control check. (DOCX 208 kb) [file 12864_2018_4498_MOESM11_ESM.docx]

**Additional Table 4.** Description of sequencing quality control parameters and statistics.

| **Study number** | **Clean reads** | **Q20%** | **Total bases** | **Mean coverage** | **Median coverage** | **Percentage bases >1** |
| --- | --- | --- | --- | --- | --- | --- |
| 100865 | 5393826 | 94,86 | 496846191 | 112,60 | 117 | 99,00 |
| 100866 | 2806938 | 95,62 | 258749297 | 58,70 | 61 | 99,10 |
| 100870 | 3469296 | 94,54 | 320142664 | 72,60 | 75 | 99,20 |
| 100874 | 8497168 | 95,62 | 783313115 | 177,60 | 184 | 99,70 |
| 100876 | 6903240 | 95,75 | 636549074 | 144,30 | 150 | 99,50 |
| 100878 | 8828962 | 95,59 | 814420068 | 184,60 | 191 | 99,50 |
| 100879 | 4471336 | 95,48 | 412040142 | 93,40 | 97 | 99,00 |
| 100883 | 11819814 | 95,73 | 1089505119 | 247,00 | 255 | 99,20 |
| 100884 | 3347584 | 95,08 | 309679647 | 70,20 | 73 | 99,50 |
| 100886 | 7686658 | 95,78 | 709079468 | 160,70 | 167 | 99,20 |
| 100887 | 4668126 | 95,28 | 430856934 | 97,70 | 101 | 99,40 |
| 100890 | 8814420 | 95,67 | 814649331 | 184,70 | 192 | 99,20 |
| 100891 | 5695014 | 95,52 | 524832508 | 119,00 | 123 | 99,10 |
| 100892 | 2735174 | 95,89 | 251826716 | 57,10 | 59 | 99,00 |
| 100894 | 6729510 | 95,83 | 620328102 | 140,60 | 145 | 99,50 |
| 100895 | 9433682 | 95,74 | 871716597 | 197,60 | 205 | 99,20 |
| 100896 | 8746936 | 95,72 | 806976809 | 182,90 | 190 | 99,80 |
| 100901 | 4186882 | 95,06 | 386788589 | 87,70 | 91 | 99,20 |
| 100904 | 8058216 | 95,56 | 744905643 | 168,90 | 175 | 99,40 |
| 100906 | 5458790 | 95,47 | 455994928 | 103,40 | 107 | 99,30 |
| 100907 | 9489138 | 95,51 | 876888230 | 198,80 | 207 | 98,70 |
| 100912 | 4186690 | 94,61 | 383674366 | 87,00 | 89 | 99,20 |
| 100913 | 6683380 | 94,67 | 616012210 | 139,60 | 146 | 98,80 |
| 100917 | 5527220 | 95,57 | 508711705 | 115,30 | 119 | 99,90 |
| 100918 | 6806576 | 94,53 | 628084709 | 142,40 | 148 | 98,80 |
| 100921 | 3123650 | 95,05 | 288755589 | 65,50 | 68 | 99,50 |
| 100923 | 10913652 | 94,92 | 1007150244 | 228,30 | 238 | 99,40 |
| 100925 | 8613960 | 94,75 | 795469596 | 180,30 | 188 | 98,80 |
| 100926 | 9149536 | 95,67 | 832817109 | 188,80 | 195 | 99,30 |
| 100927 | 7666522 | 95,70 | 707747568 | 160,40 | 165 | 99,90 |
| 100928 | 5247660 | 95,03 | 483371397 | 109,60 | 114 | 98,90 |
| 100929 | 6371238 | 95,54 | 587360512 | 133,10 | 138 | 99,00 |
| 100930 | 7564418 | 95,32 | 698275127 | 158,30 | 163 | 99,50 |
| 100931 | 9221108 | 94,85 | 852549574 | 193,30 | 202 | 99,20 |
| 100932 | 6497346 | 94,98 | 599473298 | 135,90 | 142 | 99,20 |
| 100933 | 6973496 | 94,98 | 642622186 | 145,70 | 152 | 99,40 |
| 100935 | 2267830 | 94,46 | 205539485 | 46,60 | 45 | 99,30 |
| 100941 | 2677256 | 95,41 | 244781931 | 55,50 | 54 | 99,10 |
| 100943 | 3170650 | 94,63 | 292561071 | 66,30 | 69 | 98,90 |
| 100946 | 5172654 | 94,71 | 477425025 | 108,20 | 113 | 98,90 |
| 100947 | 8855840 | 95,02 | 816620961 | 185,10 | 193 | 99,20 |
| 100951 | 2767780 | 95,39 | 255056580 | 57,80 | 57 | 99,10 |
| 100960 | 4637948 | 95,43 | 425928568 | 96,60 | 99 | 99,10 |
| 100962 | 4310596 | 95,63 | 392051064 | 88,90 | 91 | 99,20 |
| 100963 | 1974408 | 94,21 | 182390070 | 41,30 | 43 | 98,80 |
| 100964 | 2262778 | 94,91 | 208683478 | 47,30 | 48 | 99,30 |
| 100967 | 2711596 | 95,22 | 248967022 | 56,40 | 58 | 99,40 |
| 100968 | 5123952 | 95,30 | 471987706 | 107,00 | 110 | 99,00 |
| 100972 | 7477410 | 95,21 | 689739641 | 156,40 | 161 | 99,20 |
| **Study number** | **Clean reads** | **Q20%** | **Total bases** | **Mean coverage** | **Median coverage** | **Percentage bases >1** |
| 100975 | 7998754 | 95,34 | 738615844 | 167,40 | 173 | 99,50 |
| 100976 | 3638594 | 94,75 | 335773295 | 76,10 | 80 | 98,80 |
| 100983 | 3669988 | 94,48 | 339045575 | 76,90 | 80 | 98,80 |
| 100984 | 6956916 | 95,35 | 641494402 | 145,40 | 145 | 99,40 |
| 100987 | 2629362 | 95,11 | 243150452 | 55,10 | 57 | 99,10 |
| 100989 | 11563038 | 95,77 | 1065686827 | 241,60 | 249 | 99,10 |
| 100991 | 6316160 | 94,96 | 582725768 | 132,10 | 138 | 98,90 |
| 100992 | 2859276 | 94,60 | 263619555 | 59,80 | 62 | 98,50 |
| 100993 | 8231110 | 95,79 | 758381553 | 171,90 | 178 | 99,20 |
| 100994 | 5536364 | 94,88 | 510337890 | 115,70 | 121 | 98,80 |
| 100996 | 7075788 | 95,07 | 651919072 | 147,80 | 153 | 99,20 |
| 100997 | 2132396 | 94,05 | 197331995 | 44,70 | 47 | 98,40 |
| 100999 | 6085482 | 95,58 | 556429986 | 126,10 | 129 | 99,60 |
| 900077 | 4657458 | 95,37 | 430691102 | 97,63 | 103 | 98,40 |
| 900083 | 6691044 | 95,56 | 617998403 | 140,09 | 147 | 98,20 |
| 900084 | 3248492 | 95,18 | 299922817 | 67,99 | 71 | 98,30 |
| 900089 | 3523302 | 95,20 | 324308817 | 73,51 | 76 | 99,30 |
| 900094 | 3387992 | 95,47 | 312171242 | 70,76 | 73 | 99,20 |
| 900095 | 4466868 | 94,91 | 411820079 | 93,40 | 94 | 99,10 |
| 900104 | 10781194 | 95,73 | 996184664 | 225,80 | 234 | 99,10 |
| 900110 | 6960366 | 96,63 | 642971426 | 145,75 | 150 | 99,80 |
| 900115 | 5065176 | 95,64 | 466750089 | 105,80 | 110 | 98,80 |
| 900126 | 4039714 | 95,29 | 373033196 | 84,56 | 89 | 98,00 |
| 900130 | 8172166 | 94,80 | 755611366 | 171,30 | 175 | 99,00 |
| 900135 | 6004426 | 95,27 | 555768696 | 126,00 | 130 | 99,30 |
| 900138 | 7583758 | 95,44 | 700928339 | 158,90 | 165 | 99,20 |
| 900144 | 4087780 | 95,28 | 376924265 | 85,44 | 88 | 99,10 |
| 900150 | 6564174 | 95,46 | 604493321 | 137,03 | 143 | 98,50 |
| 900157 | 13620988 | 95,57 | 1261781723 | 286,02 | 296 | 99,00 |
| 900159 | 5053700 | 95,72 | 465523074 | 105,50 | 109 | 99,00 |
| 900160 | 791534 | 94,83 | 72810185 | 16,50 | 17 | 98,90 |
| 900161 | 6767554 | 95,40 | 622483695 | 141,10 | 146 | 99,50 |
| 900164 | 5978184 | 94,93 | 551296262 | 125,00 | 130 | 99,00 |
| 900166 | 8463916 | 94,68 | 783004573 | 177,50 | 184 | 98,70 |
| 900169 | 3531234 | 95,09 | 324288155 | 73,50 | 73 | 99,20 |
| 900173 | 4865874 | 94,96 | 448459089 | 101,70 | 105 | 99,10 |
| 900174 | 6456632 | 94,66 | 596399079 | 135,20 | 140 | 98,80 |
| 900176 | 5891534 | 94,74 | 545263881 | 123,60 | 129 | 98,60 |
| 900177 | 8367724 | 95,47 | 769016835 | 174,30 | 180 | 99,10 |
| 900178 | 5884278 | 94,75 | 544283139 | 123,40 | 128 | 98,80 |
| 900179 | 3757352 | 94,53 | 346790136 | 78,60 | 82 | 98,50 |
| 900181 | 3658838 | 94,80 | 338192325 | 76,66 | 80 | 98,30 |
| 900183 | 4112656 | 94,50 | 379449608 | 86,00 | 89 | 99,50 |
| 900190 | 5386042 | 95,48 | 496968260 | 112,65 | 117 | 99,60 |
| 900192 | 4411450 | 96,52 | 407585797 | 92,39 | 95 | 99,60 |
| 900199 | 9405206 | 95,68 | 869958388 | 197,20 | 204 | 99,30 |
| 900201 | 2187488 | 95,12 | 201682848 | 45,70 | 47 | 99,10 |
| 900202 | 8860806 | 95,83 | 818983627 | 185,70 | 192 | 99,20 |
| 900204 | 4196972 | 94,37 | 387936608 | 87,90 | 92 | 98,50 |
| 900206 | 6629018 | 94,70 | 613385999 | 139,00 | 145 | 98,60 |
| 900211 | 8882108 | 95,37 | 819024355 | 185,70 | 191 | 99,10 |
| **Study number** | **Clean reads** | **Q20%** | **Total bases** | **Mean coverage** | **Median coverage** | **Percentage bases >1** |
| 900212 | 8926712 | 95,66 | 822954141 | 186,60 | 193 | 99,00 |
| 900215 | 6848790 | 95,51 | 630580535 | 142,90 | 147 | 99,10 |
| 900221 | 2812730 | 95,25 | 260233372 | 58,99 | 62 | 98,30 |
| 900223 | 3083040 | 95,31 | 284289254 | 64,44 | 67 | 99,10 |
| 900225 | 4983966 | 95,07 | 460812198 | 104,50 | 108 | 99,50 |
| 900229 | 6520394 | 94,75 | 601645866 | 136,40 | 142 | 98,80 |
| 900236 | 12376840 | 96,27 | 1127089142 | 255,50 | 261 | 99,70 |
| 900238 | 9682506 | 95,50 | 895230730 | 202,93 | 213 | 98,30 |
| 900239 | 6366596 | 96,72 | 587033189 | 133,07 | 137 | 99,30 |
| 900241 | 5696554 | 96,58 | 527413573 | 119,55 | 123 | 99,40 |
| 900244 | 7550754 | 95,37 | 696142653 | 157,80 | 163 | 99,10 |
| 900251 | 5570528 | 95,79 | 513273278 | 116,40 | 120 | 99,30 |
| 900319 | 4327172 | 95,17 | 393895196 | 89,29 | 88 | 99,20 |
| 900343 | 4226624 | 95,62 | 390290590 | 88,47 | 93 | 97,90 |
| 900344 | 7305994 | 94,54 | 673506466 | 152,70 | 159 | 98,80 |
| 900365 | 3633014 | 95,68 | 336204924 | 76,21 | 80 | 98,40 |
| 900374 | 7682274 | 95,54 | 709433935 | 160,81 | 169 | 98,10 |
| 900387 | 4761330 | 95,25 | 438801669 | 99,50 | 99 | 99,30 |
| 900394 | 5895104 | 95,17 | 545589186 | 123,70 | 128 | 99,10 |
| 900397 | 6781232 | 95,65 | 626276164 | 141,96 | 149 | 98,50 |
| 900400 | 7612354 | 95,39 | 703612915 | 159,49 | 167 | 98,20 |
| 900406 | 7457526 | 95,41 | 685335643 | 155,40 | 159 | 99,20 |
| 900413 | 8074132 | 94,82 | 747137218 | 169,40 | 177 | 98,70 |
| 900416 | 5346728 | 94,62 | 494699921 | 112,10 | 117 | 98,70 |
| 900417 | 3282346 | 94,57 | 302572231 | 68,60 | 72 | 98,30 |
| 900421 | 6792232 | 94,41 | 628043107 | 142,40 | 149 | 98,60 |
| 900428 | 6919752 | 94,64 | 640814487 | 145,30 | 153 | 98,00 |
| 900430 | 4576412 | 95,83 | 421902959 | 95,60 | 99 | 98,80 |
| 900437 | 10100234 | 94,68 | 932826377 | 211,50 | 220 | 98,40 |
| 900439 | 1719270 | 95,22 | 158735817 | 35,98 | 38 | 97,90 |
| 900443 | 6746410 | 95,67 | 622308095 | 141,10 | 146 | 99,30 |
| 900446 | 3568466 | 95,49 | 328026972 | 74,40 | 76 | 99,10 |
| 900459 | 3350726 | 94,33 | 309370206 | 70,10 | 73 | 98,70 |
| 900464 | 10104002 | 95,89 | 931474859 | 211,20 | 216 | 99,10 |
| 900469 | 5572320 | 95,63 | 514346967 | 116,60 | 120 | 99,60 |
| 900477 | 10793432 | 94,58 | 996500701 | 225,90 | 236 | 98,60 |
| 900489 | 5893196 | 95,85 | 543161841 | 123,10 | 128 | 98,80 |
| 900490 | 5546714 | 95,61 | 512275952 | 116,12 | 122 | 98,20 |
| 900517 | 5739918 | 94,60 | 531133943 | 120,40 | 126 | 98,60 |
| 900573 | 7510088 | 95,56 | 693375210 | 157,17 | 165 | 98,60 |
| 900574 | 7482078 | 95,25 | 688955525 | 156,17 | 162 | 99,10 |
| 900576 | 9422310 | 95,75 | 868555554 | 196,90 | 203 | 98,80 |
| 900580 | 2745100 | 95,70 | 232761189 | 52,80 | 54 | 99,00 |
| 900586 | 2581044 | 95,48 | 238334139 | 54,03 | 57 | 98,00 |
| 900601 | 9062300 | 95,53 | 836943089 | 189,72 | 199 | 98,20 |
| 900603 | 6675866 | 95,62 | 616819650 | 139,80 | 145 | 99,20 |
| 900609 | 4064352 | 94,30 | 375056975 | 85,00 | 89 | 98,30 |
| 900611 | 6260624 | 95,40 | 577124778 | 130,80 | 135 | 99,30 |
| 900612 | 5889964 | 95,58 | 542995499 | 123,10 | 127 | 99,00 |
| 900618 | 5136100 | 95,46 | 473770925 | 107,40 | 110 | 99,10 |
| 900637 | 2483164 | 95,21 | 229386411 | 52,00 | 54 | 99,20 |
| **Study number** | **Clean reads** | **Q20%** | **Total bases** | **Mean coverage** | **Median coverage** | **Percentage bases >1** |
| 900639 | 8184572 | 95,33 | 757111404 | 171,60 | 177 | 99,30 |
| 900654 | 4897320 | 95,37 | 450923632 | 102,20 | 105 | 99,30 |
| 900655 | 3036462 | 94,58 | 280487243 | 63,60 | 67 | 98,70 |
| 900666 | 5199786 | 95,85 | 478841323 | 108,50 | 112 | 99,00 |
| 900677 | 3880296 | 94,56 | 358103680 | 81,20 | 85 | 98,60 |
| 900680 | 5220468 | 94,67 | 481344407 | 109,10 | 114 | 98,70 |
| 900703 | 2435754 | 94,78 | 225517592 | 51,10 | 53 | 98,60 |
| 900706 | 4981156 | 95,53 | 461099564 | 104,52 | 109 | 98,20 |
| 900711 | 4677424 | 94,92 | 430474568 | 97,60 | 99 | 99,30 |
| 900716 | 10183442 | 94,59 | 938814696 | 212,80 | 222 | 98,80 |
| 900731 | 5091556 | 95,37 | 470191118 | 106,58 | 112 | 98,00 |
| 900746 | 6474714 | 95,36 | 598086075 | 135,57 | 142 | 98,10 |
| 900748 | 6584354 | 95,10 | 607985996 | 137,82 | 144 | 98,40 |
| 900751 | 3003642 | 95,37 | 276844453 | 62,75 | 65 | 99,00 |
| 900824 | 6534112 | 96,35 | 578749267 | 131,20 | 131 | 99,40 |
| 900845 | 3141548 | 94,36 | 290278154 | 65,80 | 69 | 98,60 |
| 900849 | 4992552 | 95,91 | 460361996 | 104,40 | 108 | 99,30 |
| 1001002 | 8421510 | 94,85 | 779007911 | 176,60 | 184 | 99,20 |
| 1001003 | 3107372 | 95,52 | 286077988 | 64,90 | 67 | 99,10 |
| 1001009 | 4275054 | 95,13 | 394554574 | 89,40 | 93 | 99,30 |
| 1001011 | 5828312 | 94,85 | 537712611 | 121,90 | 127 | 99,30 |
| 1001012 | 5244854 | 95,36 | 483589099 | 109,60 | 113 | 99,60 |
| 1001013 | 8923730 | 95,41 | 822488632 | 186,40 | 193 | 99,70 |
| 1100213 | 4266778 | 94,92 | 394750818 | 89,48 | 93 | 98,90 |
| 1100217 | 5838902 | 95,61 | 539199148 | 122,22 | 125 | 98,80 |
| 1100231 | 4172814 | 95,42 | 384740674 | 87,21 | 90 | 98,90 |
| 1100232 | 6931214 | 95,41 | 637630282 | 144,54 | 149 | 99,40 |
| 1100233 | 6438244 | 96,59 | 591744287 | 134,14 | 136 | 99,60 |
| 1100234 | 2448826 | 95,04 | 226034251 | 51,24 | 54 | 97,90 |
| 1100238 | 5349728 | 95,45 | 493746431 | 111,92 | 115 | 99,20 |
| 1100246 | 4241148 | 94,92 | 392868330 | 89,05 | 92 | 99,00 |
| 1100250 | 5686092 | 95,38 | 525154268 | 119,04 | 125 | 98,10 |
| 1100254 | 691128 | 95,19 | 63775796 | 14,46 | 15 | 97,50 |
| 1100256 | 6861744 | 95,09 | 635722606 | 144,10 | 151 | 98,50 |
| 1100262 | 1867970 | 92,89 | 172516103 | 39,11 | 41 | 98,90 |
| 1100264 | 8528690 | 95,17 | 788677462 | 178,78 | 185 | 99,20 |
| 1100269 | 5137284 | 95,01 | 474920172 | 107,65 | 112 | 99,10 |
| 1100270 | 6399968 | 95,37 | 589507896 | 133,63 | 138 | 99,00 |
| 1100273 | 6762182 | 95,34 | 625911758 | 141,88 | 149 | 98,30 |
| 1100280 | 4627628 | 95,43 | 427362057 | 96,87 | 101 | 98,40 |
| 1100281 | 6110340 | 95,50 | 564048380 | 127,86 | 132 | 99,20 |
| 1100283 | 4638990 | 95,44 | 429132510 | 97,28 | 102 | 98,50 |
| 1100291 | 5293572 | 96,58 | 488900426 | 110,82 | 114 | 99,70 |
| 1100294 | 7450520 | 95,38 | 688696972 | 156,11 | 160 | 99,40 |
| 1100300 | 3092126 | 95,00 | 286048335 | 64,84 | 68 | 98,30 |
| 1100306 | 5766562 | 95,54 | 532393055 | 120,68 | 127 | 98,30 |
| 1100307 | 5090484 | 94,61 | 470152143 | 106,57 | 111 | 98,00 |
| 1100315 | 4590438 | 95,26 | 423829736 | 96,07 | 101 | 98,00 |
| 1100316 | 4639218 | 95,00 | 429207921 | 97,29 | 102 | 98,20 |
| 1100320 | 4273028 | 95,14 | 394124860 | 89,34 | 93 | 98,80 |
| 1100331 | 2893908 | 95,10 | 267254684 | 60,58 | 63 | 98,20 |
| **Study number** | **Clean reads** | **Q20%** | **Total bases** | **Mean coverage** | **Median coverage** | **Percentage bases >1** |
| 1100332 | 3547096 | 95,38 | 327284397 | 74,19 | 76 | 99,10 |
| 1100333 | 7640658 | 95,53 | 702530395 | 159,25 | 164 | 99,40 |
| 1100335 | 4719994 | 95,23 | 436845013 | 99,02 | 104 | 98,10 |
| 1100340 | 10582420 | 95,47 | 977856076 | 221,66 | 232 | 98,60 |
| 1100345 | 5664472 | 95,22 | 523635379 | 118,70 | 124 | 98,50 |
| 1100348 | 9478592 | 96,71 | 874252406 | 198,17 | 204 | 99,20 |
| 1100353 | 7157040 | 96,90 | 662329285 | 150,14 | 155 | 99,60 |
| 1100367 | 4378604 | 96,73 | 403022547 | 91,36 | 94 | 99,40 |
| 1100369 | 3614860 | 95,09 | 334572793 | 75,84 | 79 | 98,40 |
| 1100373 | 7817932 | 96,89 | 721194175 | 163,48 | 169 | 99,30 |
| 1100374 | 5687648 | 95,12 | 524404441 | 118,87 | 123 | 99,30 |
| 1100396 | 4464604 | 96,87 | 412472505 | 93,50 | 96 | 99,60 |
| 1100397 | 7952458 | 95,27 | 734301221 | 166,45 | 172 | 99,10 |
| 1100400 | 4156544 | 94,85 | 384729079 | 87,21 | 91 | 98,50 |
| 1100421 | 8161636 | 96,93 | 744477220 | 168,76 | 174 | 99,30 |
| 1100607 | 6876462 | 95,38 | 632158432 | 143,30 | 148 | 99,40 |
| 1100707 | 4391668 | 95,52 | 405964237 | 92,02 | 96 | 98,90 |
| 1100708 | 5804762 | 95,37 | 535042353 | 121,28 | 126 | 98,90 |
| 1100716 | 8686226 | 95,58 | 800338659 | 181,42 | 188 | 98,80 |
| 1100730 | 8065486 | 96,76 | 746592857 | 169,24 | 174 | 99,70 |
| 1100738 | 5401532 | 95,40 | 500478912 | 113,45 | 118 | 98,30 |
| 1100746 | 3480194 | 95,27 | 321712017 | 72,93 | 75 | 98,80 |
| 1100757 | 6216226 | 95,37 | 572081247 | 129,68 | 130 | 98,90 |
| 1100760 | 3239756 | 95,38 | 298617464 | 67,69 | 71 | 98,50 |
| 1100763 | 5502764 | 95,21 | 506670287 | 114,85 | 117 | 98,70 |
| 1100764 | 5650758 | 96,92 | 521338278 | 118,18 | 122 | 99,30 |
| 1100773 | 5049028 | 95,31 | 466844260 | 105,82 | 109 | 99,10 |
| 1100774 | 1121332 | 95,44 | 103410546 | 23,44 | 25 | 98,50 |
| 1100786 | 5043614 | 95,42 | 463788799 | 105,13 | 109 | 99,10 |
| 1100797 | 6867872 | 95,63 | 631656757 | 143,18 | 147 | 99,30 |
| 1100903 | 3940012 | 95,42 | 363569155 | 82,41 | 85 | 98,60 |
| 1100907 | 6662502 | 96,74 | 616858915 | 139,83 | 144 | 99,40 |
| 1100908 | 4866202 | 95,41 | 447414934 | 101,42 | 101 | 99,00 |
| 1100909 | 4321576 | 95,18 | 398668836 | 90,37 | 94 | 98,70 |
| 1100917 | 1474708 | 92,91 | 136437753 | 30,93 | 32 | 98,40 |
| 1100918 | 8666842 | 95,55 | 800281865 | 181,41 | 190 | 98,40 |
| 1100921 | 5637528 | 96,81 | 521923240 | 118,31 | 122 | 99,60 |
| 1100929 | 4545226 | 95,44 | 419603339 | 95,12 | 98 | 99,20 |
| 1100930 | 3966012 | 95,49 | 367144208 | 83,22 | 87 | 98,30 |
| 1100931 | 4305168 | 95,24 | 398774103 | 90,39 | 94 | 99,40 |
| 1100933 | 1616234 | 92,92 | 149443084 | 33,88 | 35 | 98,40 |
| 1100936 | 4857908 | 95,07 | 448087877 | 101,57 | 105 | 98,60 |
| 1100937 | 4023822 | 95,29 | 371528087 | 84,22 | 86 | 98,90 |
| 1100942 | 3877632 | 95,55 | 357512894 | 81,04 | 84 | 98,70 |
| 1100943 | 4256638 | 95,07 | 391629990 | 88,77 | 92 | 99,00 |
| 1100945 | 3707196 | 95,44 | 342392133 | 77,61 | 81 | 98,50 |
| 1100969 | 9138662 | 95,44 | 843500045 | 191,20 | 197 | 99,20 |
| 1100977 | 5068004 | 95,36 | 467957429 | 106,08 | 111 | 98,20 |
| 1100981 | 5409442 | 95,53 | 499060319 | 113,13 | 117 | 98,70 |
| 1100984 | 5795768 | 95,60 | 535918638 | 121,48 | 126 | 99,00 |
| 1100987 | 2431690 | 96,80 | 392597634 | 88,99 | 92 | 99,00 |
| **Study number** | **Clean reads** | **Q20%** | **Total bases** | **Mean coverage** | **Median coverage** | **Percentage bases >1** |
| 1100995 | 4298726 | 95,24 | 397765175 | 90,16 | 93 | 99,10 |
| 1101003 | 8605192 | 95,75 | 793618170 | 179,90 | 186 | 99,20 |
| 1101004 | 5345552 | 95,18 | 493292026 | 111,82 | 116 | 99,20 |
| 1101008 | 6612834 | 96,66 | 610379624 | 138,36 | 142 | 99,30 |
| 1101011 | 5311910 | 95,44 | 490949034 | 111,29 | 115 | 98,90 |
| 1101012 | 4300000 | 95,19 | 397980899 | 90,21 | 93 | 98,90 |
| 1101015 | 8385930 | 95,79 | 774182342 | 175,49 | 179 | 98,90 |
| 1101018 | 2563844 | 95,70 | 237182128 | 53,76 | 56 | 98,80 |
| 1101025 | 9120244 | 95,95 | 843597287 | 191,23 | 198 | 98,90 |
| 1101028 | 1896992 | 95,80 | 175609777 | 39,81 | 41 | 98,80 |
| 1101037 | 5087396 | 95,77 | 471034289 | 106,77 | 111 | 99,20 |
| 1101047 | 7861958 | 95,42 | 727939191 | 165,01 | 173 | 98,30 |
| 1101054 | 7246156 | 95,44 | 670754167 | 152,05 | 159 | 98,40 |
| 1101061 | 8923668 | 96,99 | 823426144 | 186,65 | 193 | 99,30 |
| 1101064 | 4408312 | 95,68 | 406658628 | 92,18 | 95 | 98,50 |
| 1101067 | 8193818 | 96,70 | 756937816 | 171,58 | 177 | 99,60 |
| 1101074 | 3883520 | 95,62 | 358280741 | 81,21 | 85 | 98,50 |
| 1101077 | 3228516 | 95,68 | 298974274 | 67,77 | 70 | 98,70 |
| 1101080 | 4911704 | 95,80 | 453246251 | 102,74 | 107 | 98,70 |
| 1101084 | 5692096 | 95,77 | 525457900 | 119,11 | 124 | 98,60 |
| 1101085 | 4265798 | 94,95 | 394010661 | 89,31 | 93 | 98,80 |
| 1101088 | 3440936 | 95,98 | 318639444 | 72,23 | 75 | 98,70 |
| 1101089 | 6002548 | 96,96 | 556190844 | 126,08 | 130 | 99,60 |
| 1101253 | 3642584 | 95,74 | 336144308 | 76,20 | 79 | 98,80 |
| 1101292 | 6082440 | 95,86 | 560795868 | 127,12 | 132 | 98,80 |
| 1101303 | 9312806 | 95,99 | 861349125 | 195,25 | 202 | 99,20 |
| 1101304 | 2045310 | 93,60 | 188641178 | 42,76 | 44 | 98,50 |
| 1101316 | 10031646 | 95,64 | 923436067 | 209,32 | 214 | 99,00 |
| 1101317 | 4281276 | 94,97 | 393297145 | 89,15 | 92 | 99,00 |
| 1101318 | 8311646 | 95,94 | 767350601 | 173,94 | 180 | 99,10 |
| 1101319 | 5606644 | 95,88 | 518165835 | 117,46 | 122 | 99,00 |
| 1101321 | 13908338 | 95,81 | 1283563813 | 290,96 | 303 | 99,20 |
| 1101322 | 5388084 | 95,89 | 496954407 | 112,65 | 117 | 98,70 |
| 1101323 | 4780336 | 95,88 | 441188685 | 100,01 | 103 | 98,80 |
| 1101324 | 10556482 | 96,05 | 974353093 | 220,87 | 228 | 99,20 |
| 1101329 | 5265746 | 95,54 | 485933498 | 110,15 | 114 | 98,60 |
| 1101337 | 9036512 | 95,73 | 834483421 | 189,16 | 196 | 98,90 |
| 1101340 | 5004558 | 95,76 | 456531484 | 103,49 | 102 | 99,00 |
| 1101342 | 8516402 | 95,68 | 787938504 | 178,61 | 185 | 99,00 |
| 1101344 | 5945098 | 95,92 | 548757919 | 124,39 | 129 | 98,90 |
| 1101346 | 5892778 | 95,82 | 541723597 | 122,80 | 127 | 98,90 |
| 1101348 | 7181066 | 95,79 | 662390301 | 150,15 | 156 | 98,80 |
| 1101350 | 5585560 | 95,70 | 517096265 | 117,21 | 122 | 98,80 |
| 1101355 | 5142926 | 95,59 | 475691952 | 107,83 | 112 | 98,70 |
| 1101359 | 8357208 | 95,68 | 769440613 | 174,42 | 180 | 99,00 |
| 1101360 | 6005832 | 95,75 | 554392328 | 125,67 | 130 | 99,10 |
| 1101367 | 2839034 | 95,73 | 262799618 | 59,57 | 62 | 98,80 |
| 1101368 | 5295318 | 95,94 | 488701549 | 110,78 | 115 | 98,40 |
| 1101382 | 2494612 | 95,72 | 229415793 | 52,00 | 54 | 98,50 |
| 1101386 | 9713036 | 95,80 | 896253841 | 203,16 | 211 | 99,10 |
| 1101554 | 8768224 | 95,99 | 809038817 | 183,39 | 190 | 99,00 |
| **Study number** | **Clean reads** | **Q20%** | **Total bases** | **Mean coverage** | **Median coverage** | **Percentage bases >1** |
| 1101556 | 2695552 | 93,65 | 248575069 | 56,35 | 58 | 98,30 |
| 1101557 | 5974612 | 95,54 | 552272734 | 125,19 | 130 | 98,80 |
| 1101572 | 9706246 | 95,94 | 895520927 | 203,00 | 211 | 98,80 |
| 1101576 | 4786726 | 95,86 | 441905771 | 100,17 | 104 | 98,70 |
| 1101580 | 7791782 | 95,82 | 718652884 | 162,90 | 169 | 98,90 |
| 1101600 | 3010808 | 95,87 | 278389255 | 63,10 | 65 | 98,70 |
| 1101602 | 3259454 | 95,92 | 299698944 | 67,94 | 71 | 98,80 |
| 1101603 | 7436708 | 96,04 | 686272907 | 155,56 | 161 | 98,90 |
| 1101604 | 6303476 | 95,18 | 581855643 | 131,89 | 136 | 98,90 |
| 1101610 | 5124546 | 96,59 | 472976032 | 107,21 | 111 | 99,30 |
| 1101623 | 5027232 | 95,51 | 463403211 | 105,04 | 108 | 98,70 |
| 1101628 | 5667660 | 95,74 | 524058595 | 118,79 | 123 | 99,10 |
| 1101652 | 7220436 | 96,62 | 666135610 | 151,00 | 156 | 99,30 |
| 1101933 | 4298652 | 95,27 | 397732878 | 90,16 | 93 | 99,10 |
| 1101942 | 4273636 | 96,65 | 395475578 | 89,65 | 92 | 99,60 |
| 1101947 | 5441952 | 96,70 | 499732433 | 113,28 | 115 | 99,30 |
| 1101984 | 4843912 | 96,79 | 446859913 | 101,29 | 105 | 99,20 |
| 1101997 | 5845746 | 96,62 | 538540400 | 122,08 | 126 | 99,80 |
| **Samples excluded from further analysis** | | | | | | |
| 100973 | 10916526 | 96,14 | 1042435 | 0,12 | 1 | 2,40 |
| 900089 | 6737346 | 96.70 | 289038979 | 32,76 | 34 | 99,25 |
| 900132 | 3437888 | 97.96 | 32333418 | 7,33 | 8 | 96,90 |
| 900248 | 1760232 | 96.56 | NA | NA | NA | NA |
| 900558 | 1303536 | 97.11 | NA | NA | NA | NA |
| 900625 | 5877240 | 95.53 | 1290461 | 0,29 | 1 | 10,70 |
| 1100343 | 8561266 | 95.67 | 6480416 | 1,47 | 2 | 60,60 |
| 1100772 | 1340688 | 94.87 | NA | NA | NA | NA |
| 1101001 | 8104650 | 96.05 | 275263806 | 62,30 | 65 | 98,30 |
