# Supplementary material for: Large-scale genomic analysis shows association between homoplastic genetic variation in Mycobacterium tuberculosis genes and meningeal or pulmonary tuberculosis
Source: BMC Genomics. 2018 Feb 5;19:122. doi: 10.1186/s12864-018-4498-z (PMC5800017; doi:10.1186/s12864-018-4498-z)
Supplement: Supplementary file 12 — PE / PPE genes and drug resistance genes excluded for the phylogeny construction. Listed are the genes that were excluded from the multiple alignment used to create the phylogenetic tree. (DOCX 158 kb) [file 12864_2018_4498_MOESM12_ESM.docx]

**Additional Table 5.** PE / PPE genes and drug resistance genes excluded for the

| **PE/PPE genes and genes in repetitive regions** | | | Rv2107 | Rv2741 | Rv3381c | **Known drug resistance genes** |
| --- | --- | --- | --- | --- | --- | --- |
| Rv0031 | Rv0922 | Rv1575 | Rv2108 | Rv2768c | Rv3386 |  |
| Rv0096 | Rv0977 | Rv1576c | Rv2123 | Rv2769c | Rv3387 |  |
| Rv0109 | Rv0978c | Rv1577c | Rv2126c | Rv2770c | Rv3388 |  |
| Rv0124 | Rv0980c | Rv1578c | Rv2162c | Rv2791c | Rv3425 | accD6 |
| Rv0151c | Rv1034c | Rv1579c | Rv2167c | Rv2810c | Rv3426 | ahpC |
| Rv0152c | Rv1035c | Rv1580c | Rv2168c | Rv2812 | Rv3427c | efpA |
| Rv0159c | Rv1036c | Rv1581c | Rv2177c | Rv2814c | Rv3428c | embA |
| Rv0160c | Rv1039c | Rv1582c | Rv2278 | Rv2815c | Rv3429 | embB |
| Rv0256c | Rv1040c | Rv1583c | Rv2279 | Rv2853 | Rv3430c | embC |
| Rv0278c | Rv1041c | Rv1584c | Rv2328 | Rv2885c | Rv3474 | embR |
| Rv0279c | Rv1042c | Rv1585c | Rv2340c | Rv2892c | Rv3475 | ethA |
| Rv0280 | Rv1047 | Rv1586c | Rv2352c | Rv2943 | Rv3477 | fabD |
| Rv0285 | Rv1054 | Rv1646 | Rv2353c | Rv2943A | Rv3478 | fadE24 |
| Rv0286 | Rv1067c | Rv1651c | Rv2354 | Rv2944 | Rv3507 | fbpC |
| Rv0297 | Rv1068c | Rv1705c | Rv2355 | Rv2961 | Rv3508 | furA |
| Rv0304c | Rv1087 | Rv1706c | Rv2356c | Rv2978c | Rv3511 | gid |
| Rv0305c | Rv1088 | Rv1753c | Rv2371 | Rv3018A | Rv3512 | gyrA |
| Rv0335c | Rv1089 | Rv1756c | Rv2396 | Rv3018c | Rv3514 | gyrB |
| Rv0354c | Rv1091 | Rv1757c | Rv2408 | Rv3021c | Rv3532 | inhA |
| Rv0355c | Rv1135c | Rv1763 | Rv2424c | Rv3022A | Rv3533c | iniA |
| Rv0387c | Rv1149 | Rv1764 | Rv2430c | Rv3022c | Rv3539 | iniB |
| Rv0388c | Rv1168c | Rv1765A | Rv2431c | Rv3023c | Rv3558 | iniC |
| Rv0442c | Rv1169c | Rv1768 | Rv2479c | Rv3115 | Rv3590c | kasA |
| Rv0453 | Rv1172c | Rv1787 | Rv2480c | Rv3125c | Rv3595c | katG |
| Rv0532 | Rv1195 | Rv1788 | Rv2487c | Rv3135 | Rv3621c | fabG1 |
| Rv0578c | Rv1196 | Rv1789 | Rv2490c | Rv3136 | Rv3622c | manB |
| Rv0741 | Rv1199c | Rv1790 | Rv2512c | Rv3144c | Rv3636 | ndh |
| Rv0742 | Rv1214c | Rv1791 | Rv2519 | Rv3159c | Rv3637 | nat |
| Rv0746 | Rv1243c | Rv1800 | Rv2591 | Rv3184 | Rv3638 | oxyR |
| Rv0747 | Rv1313c | Rv1801 | Rv2608 | Rv3185 | Rv3640c | pncA |
| Rv0754 | Rv1325c | Rv1802 | Rv2615c | Rv3186 | Rv3650 | rmlD |
| Rv0755A | Rv1361c | Rv1803c | Rv2634c | Rv3187 | Rv3652 | rpoB |
| Rv0755c | Rv1369c | Rv1806 | Rv2646 | Rv3191c | Rv3653 | rpsL |
| Rv0795 | Rv1370c | Rv1807 | Rv2648 | Rv3325 | Rv3738c | rrs |
| Rv0796 | Rv1386 | Rv1808 | Rv2649 | Rv3326 | Rv3739c | Rv0340 |
| Rv0797 | Rv1387 | Rv1809 | Rv2650c | Rv3327 | Rv3746c | Rv1592c |
| Rv0832 | Rv1396c | Rv1818c | Rv2651c | Rv3343c | Rv3751 | Rv1772 |
| Rv0833 | Rv1430 | Rv1840c | Rv2652c | Rv3344c | Rv3798 | Rv2242 |
| Rv0834c | Rv1441c | Rv1917c | Rv2653c | Rv3345c | Rv3812 | Rv3124 |
| Rv0850 | Rv1450c | Rv1918c | Rv2654c | Rv3347c | Rv3827c | Rv3125c |
| Rv0872c | Rv1452c | Rv1983 | Rv2655c | Rv3348 | Rv3844 | Rv3126c |
| Rv0878c | Rv1468c | Rv2013 | Rv2656c | Rv3349c | Rv3872 | thyA |
| Rv0915c | Rv1548c | Rv2014 | Rv2657c | Rv3350c | Rv3873 | tlyA |
| Rv0916c | Rv1573 | Rv2105 | Rv2659c | Rv3367 | Rv3892c | accD6 |
| Rv0920c | Rv1574 | Rv2106 | Rv2666 | Rv3380c | Rv3893c |  |

phylogeny construction.
