# Supplementary material for: Large-scale genomic analysis shows association between homoplastic genetic variation in Mycobacterium tuberculosis genes and meningeal or pulmonary tuberculosis
Source: BMC Genomics. 2018 Feb 5;19:122. doi: 10.1186/s12864-018-4498-z (PMC5800017; doi:10.1186/s12864-018-4498-z)
Supplement: Supplementary file 11 [file 12864_2018_4498_MOESM13_ESM.docx]

Predicted mutation						
**evidence**	**seq id**	**position**	**mutation**	**annotation**	**gene**	**description**
RA	NC_000962	132,417	C→G	R346G (CGC→GGC)	*PE_PGRS1* →	PE-PGRS family protein PE_PGRS1

Read alignment evidence...											
	**seq id**	**position**		**ref**	**new**	**freq**	**score (cons/poly)**	**reads**	**annotation**	**genes**	**product**
*	NC_000962	132,417	0	C	G	100.0%	201.1 / NA	60	R346G (CGC→GGC)	*PE_PGRS1*	PE-PGRS family protein PE_PGRS1
Reads supporting (aligned to +/- strand): **ref** base C (0/0); **new** base G (16/44); **total** (16/44)											

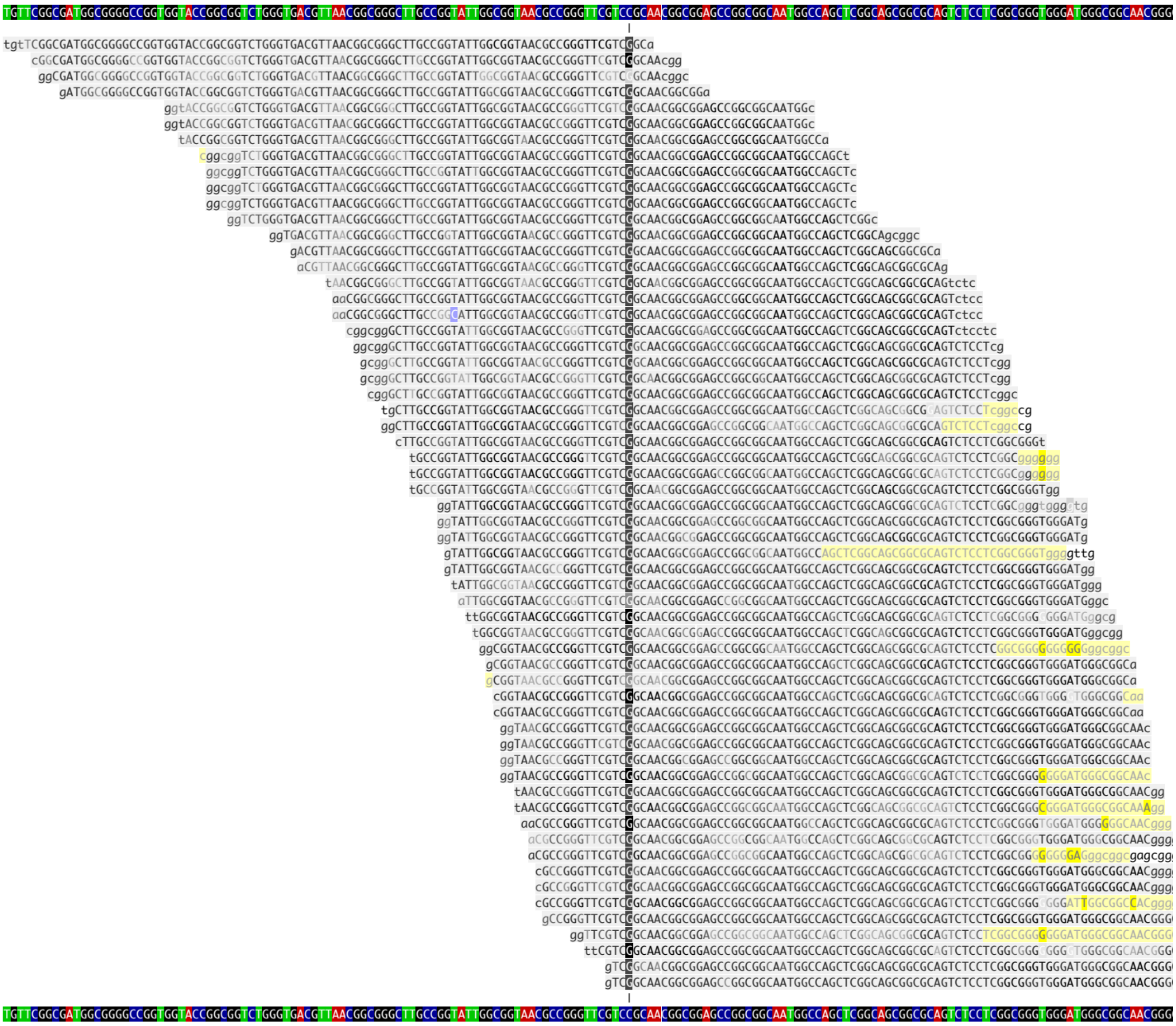

Alignment Legend

Aligned base mismatch/match (shaded by quality score): ATCG/ATCG < 3 ≤ ATCG/ATCG < 20 ≤ ATCG/ATCG < 27 ≤ ATCG/ATCG < 33 ≤ ATCG/ATCG < 41 ≤ ATCG/ATCG

Unaligned base: atcg   Masked matching base: atcg   Alignment gap: -   Deleted base: -
