# Supplementary material for: Large-scale genomic analysis shows association between homoplastic genetic variation in Mycobacterium tuberculosis genes and meningeal or pulmonary tuberculosis
Source: BMC Genomics. 2018 Feb 5;19:122. doi: 10.1186/s12864-018-4498-z (PMC5800017; doi:10.1186/s12864-018-4498-z)
Supplement: Supplementary file 12 [file 12864_2018_4498_MOESM4_ESM.docx]

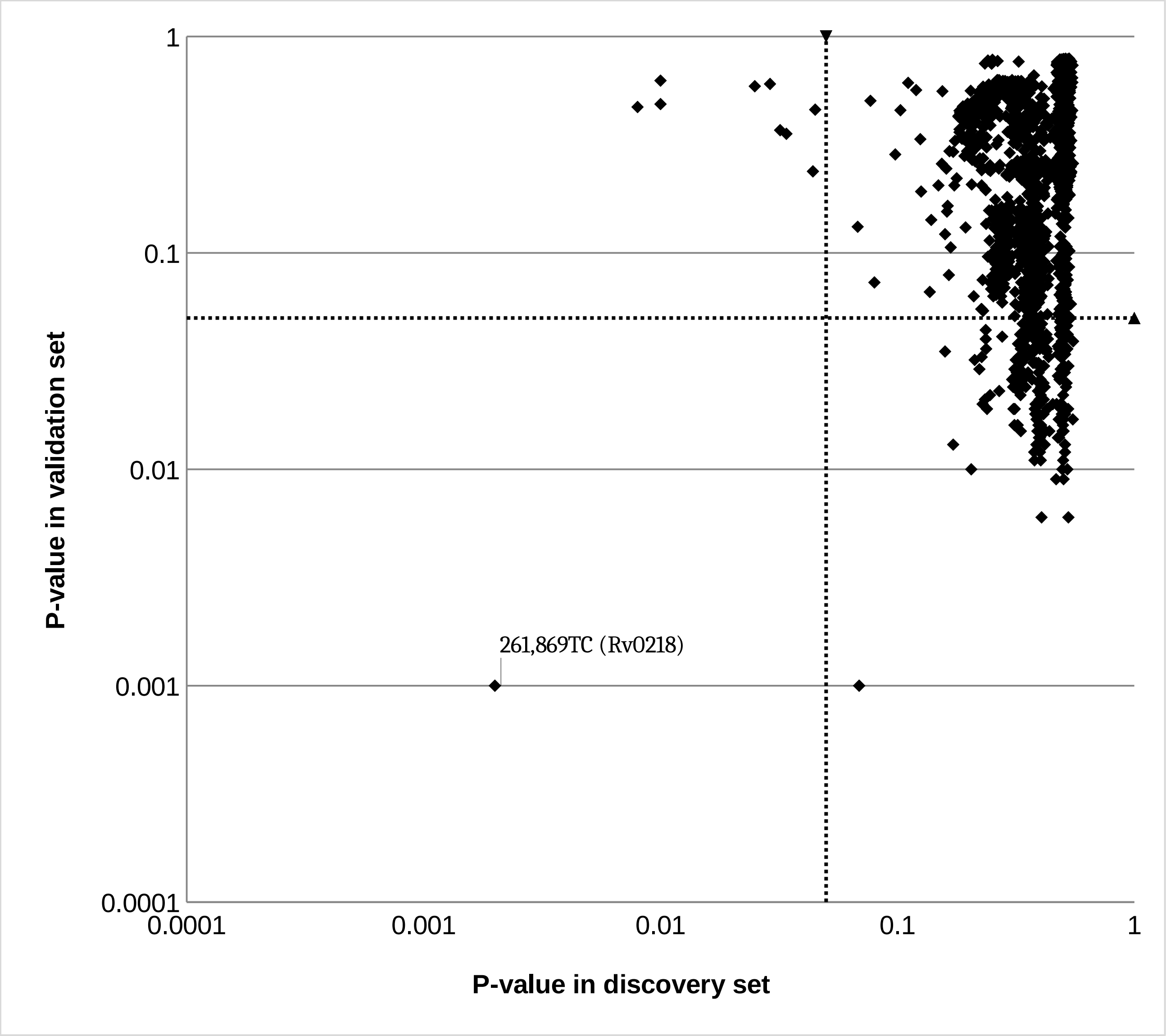

1
0.1
0.01
0.001
0.0001
0.0001
0.001
0.01
0.1
1
P-value in validation set
P-value in discovery set
261,869TC (Rv0218)
