# Supplementary material for: Large-scale genomic analysis shows association between homoplastic genetic variation in Mycobacterium tuberculosis genes and meningeal or pulmonary tuberculosis
Source: BMC Genomics. 2018 Feb 5;19:122. doi: 10.1186/s12864-018-4498-z (PMC5800017; doi:10.1186/s12864-018-4498-z)
Supplement: Supplementary file 13 [file 12864_2018_4498_MOESM5_ESM.docx]

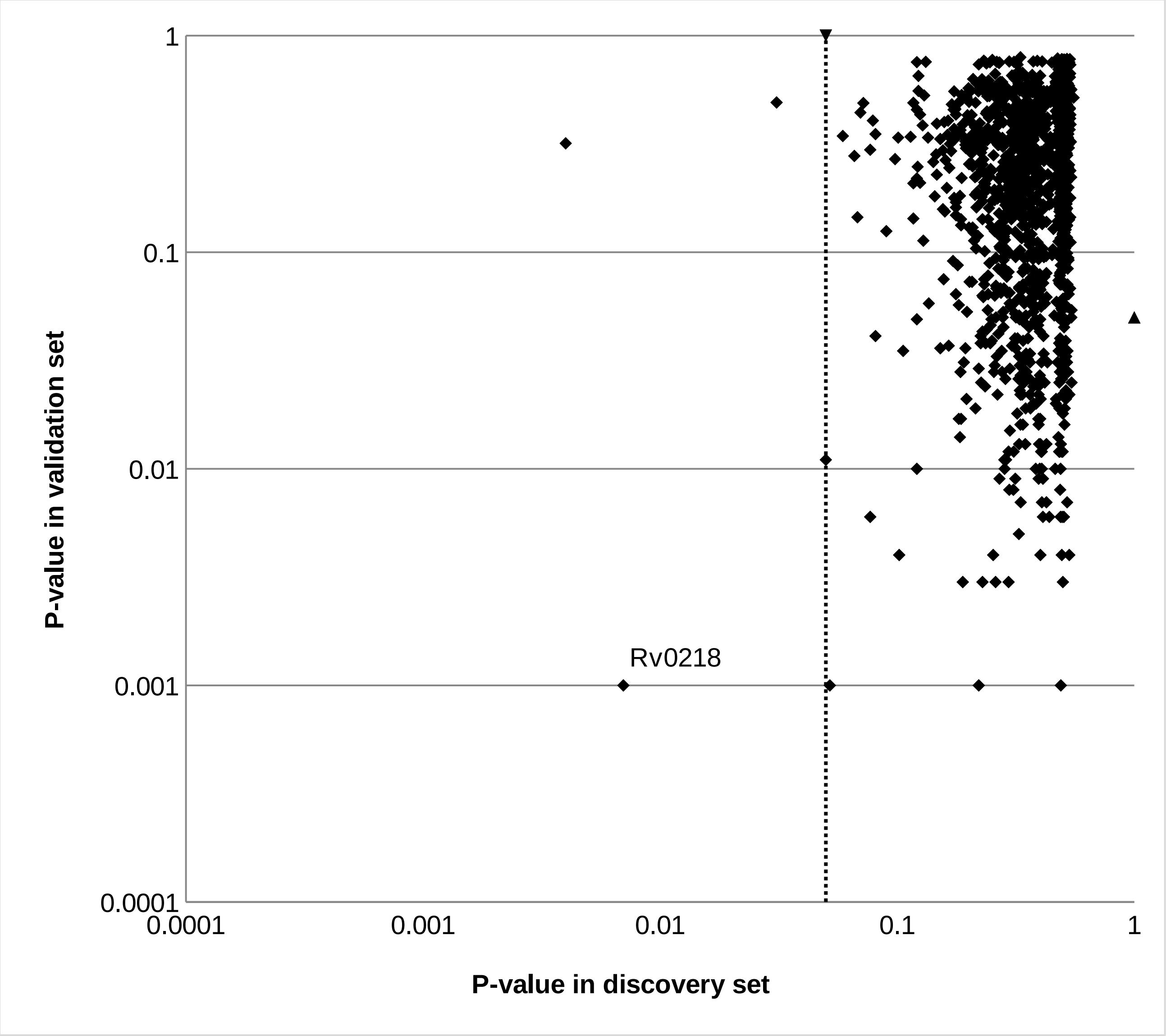
1
0.1
0.01
0.001
0.0001
P-value in validation set
Rv0218
0.0001
0.001
0.01
0.1
1
P-value in discovery set
